# Unraveling the role of M1 macrophage and CXCL9 in predicting immune checkpoint inhibitor efficacy through multicohort analysis and single‐cell RNA sequencing

**DOI:** 10.1002/mco2.471

**Published:** 2024-03-01

**Authors:** Yunfang Yu, Haizhu Chen, Wenhao Ouyang, Jin Zeng, Hong Huang, Luhui Mao, Xueyuan Jia, Taihua Guan, Zehua Wang, Ruichong Lin, Zhenjun Huang, Hanqi Yin, Herui Yao, Kang Zhang

**Affiliations:** ^1^ Faculty of Medicine Macau University of Science and Technology Macao P. R. China; ^2^ Guangdong Provincial Key Laboratory of Malignant Tumor Epigenetics and Gene Regulation Guangdong‐Hong Kong Joint Laboratory for RNA Medicine Department of Medical Oncology Breast Tumor Centre Phase I Clinical Trial Centre Sun Yat‐sen Memorial Hospital Sun Yat‐sen University Guangzhou P. R. China; ^3^ Faculty of Sustainable Development Macau University of Science and Technology Macau P. R. China; ^4^ Guangzhou National Laboratory Guangzhou P. R. China; ^5^ School of Medicine Guilin Medical University Guilin P. R. China; ^6^ Division of Science and Technology Beijing Normal University‐Hong Kong Baptist University United International College Zhuhai P. R. China; ^7^ Faculty of Innovation Engineering Macau University of Science and Technology Macao P. R. China; ^8^ South China Institute of Biomedine Guangzhou China; ^9^ Zhuhai International Eve Center Zhuhai People's Hospital and the First Affiliated Hospital of Faculty of Medicine Macau University of Science and Technology and University Hospital Zhuhai China

**Keywords:** Apolipoprotein B MRNA Editing Enzyme Catalytic Subunit 3G (APOBEC3G), C‐X‐C Motif Chemokine Ligand 9 (CXCL9), immune checkpoint inhibitors, M1 macrophage, multi‐level attention graph neural network, tumor immune microenvironment

## Abstract

The exact function of M1 macrophages and CXCL9 in forecasting the effectiveness of immune checkpoint inhibitors (ICIs) is still not thoroughly investigated. We investigated the potential of M1 macrophage and C‐X‐C Motif Chemokine Ligand 9 (CXCL9) as predictive markers for ICI efficacy, employing a comprehensive approach integrating multicohort analysis and single‐cell RNA sequencing. A significant correlation between high M1 macrophage and improved overall survival (OS) and objective response rate (ORR) was found. M1 macrophage expression was most pronounced in the immune‐inflamed phenotype, aligning with increased expression of immune checkpoints. Furthermore, CXCL9 was identified as a key marker gene that positively correlated with M1 macrophage and response to ICIs, while also exhibiting associations with immune‐related pathways and immune cell infiltration. Additionally, through exploring RNA epigenetic modifications, we identified Apolipoprotein B MRNA Editing Enzyme Catalytic Subunit 3G (APOBEC3G) as linked to ICI response, with high expression correlating with improved OS and immune‐related pathways. Moreover, a novel model based on M1 macrophage, CXCL9, and APOBEC3G‐related genes was developed using multi‐level attention graph neural network, which showed promising predictive ability for ORR. This study illuminates the pivotal contributions of M1 macrophages and CXCL9 in shaping an immune‐active microenvironment, correlating with enhanced ICI efficacy. The combination of M1 macrophage, CXCL9, and APOBEC3G provides a novel model for predicting clinical outcomes of ICI therapy, facilitating personalized immunotherapy.

## Graphical abstract

This study explores M1 macrophage and CXCL9 as predictive markers for immune checkpoint inhibitor (ICI) efficacy through multicohort analyses. High M1 macrophage correlates with improved ICI efficacy, and CXCL9 is identified as a key marker gene positively correlated with M1 macrophage and ICI efficacy. Transcriptomics and scRNA‐seq analyses unveil APOBEC3G as a novel biomarker for predicting ICI response. A novel multi‐level attention graph neural network model is proposed, showing promising performance in predicting ICI response.

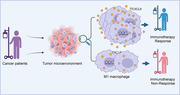

## 1 INTRODUCTION

Cancer immunotherapy, particularly immune checkpoint inhibitors (ICIs), has revolutionized the field of oncology by harnessing the immune system of patients to combat cancer.^1^ Despite the remarkable success observed in a subset of patients, a significant proportion fails to respond to ICIs, highlighting the need to identify predictive biomarkers and understand the underlying mechanisms of response and resistance.^1^ The tumor immune microenvironment (TIME) is pivotal in influencing the response to ICIs, and understanding its properties can offer significant information for categorizing patient and advancing  personalized immunotherapy.

High infiltration of CD8^+^ T cells is associated with improved responses to ICIs.^2^ Recent research has highlighted the insufficiency of T‐cell infiltration alone in identifying ICI responders.^3^ The concept of “effector immune cell deployment” has emerged, underscoring the significance of multiple immune cell (IC) types in the TIME for effective antitumor immune responses.^3^ This concept provides a rationale for transforming “cold” tumors into “hot” tumors, characterized by enhanced IC activation and infiltration.^3^ In addition to T cells, myelomonocytic cells, including macrophages, monocytes, and dendritic cells, play critical roles in antitumor immune responses.^3^ Among the various ICs in the TIME, macrophages are key players that exhibit phenotypic and functional heterogeneity. Despite substantial evidence implicating T cells in predicting ICI outcomes, the role of macrophages in this context remains poorly characterized.

Macrophages, a diverse population of ICs, exert a profound influence on the TIME and exhibit a dual nature with both pro‐ and anti‐tumorigenic effects, reflecting their plasticity in response to environmental cues.^4^, ^5^, ^6^ Deciphering the differential expression patterns and functional implications of these macrophage subtypes is crucial for comprehending their role in the context of ICIs. Recent studies have highlighted macrophages as attractive targets for checkpoint blockade therapy due to their expression of programmed cell death‐ligand 1 (PD‐L1) and PD‐L2.^5^, ^6^, ^7^ M1 macrophages, known as classically activated macrophages, are associated with pro‐inflammatory responses and have been implicated in antitumor immunity, while M2 macrophages contribute to immunosuppression within the TIME.^5^

Earlier research has suggested that M1 macrophages might act as indicators of positive responses to ICIs in individuals with metastatic urothelial cancer (mUC).^8^, ^9^, ^10^ Several cytokines and chemokines, such as those derived from macrophages in the CXCR3 chemokine system, have been identified as responsible for recruiting ICs into tumors.^11^ Accumulating evidence suggests that macrophages are major producers of the chemokine CXCL9, a ligand for the CXCR3 receptor.^9^, ^10^, ^12^, ^13^, ^14^ Macrophage‐expressed CXCL9 regulates the recruitment and localization of stem‐like CD8 T cells expressing CXCR3, which contributes to the clinical responses to anti‐programmed cell death‐1 (PD‐1) or PD‐L1 treatment.^12^ Notably, a study examining tumor‐associated macrophages in lung cancer patients revealed varying expressions of genes associated with inflammatory macrophages, specifically highlighting CXCL9, CXCL10, and Stat1 in relation to an “M1hot” phenotype.^15^ Macrophages‐derived CXCL9 correlates with increased intratumoral CD8 T‐cell density and has been demonstrated to directly enhance the function of effector CD8 T cells, thus serving as a crucial component of antitumor immunity.^9^, ^12^, ^14^ Furthermore, the epigenetic modification of RNA has been discovered to regulate immune responses through macrophage reprogramming.^16^ Nevertheless, the molecular mechanisms underlying the TIME associated with cancer immunotherapy are intricate and require further elucidation. The precise role of M1 macrophage and their molecular characteristics in predicting ICI efficacy remains poorly understood.

In this study, we aimed to unravel the role of M1 macrophage and CXCL9, a chemokine known to attract cytotoxic T cells, in predicting ICI efficacy through a multicohort analysis and single cell RNA‐sequencing (scRNA‐seq). Additionally, we investigated the interaction between M1 macrophage and other IC populations within the TIME to gain insights into the underlying mechanisms of ICI response and resistance. Furthermore, we validated our findings using independent cohorts of ICI‐treated patients and evaluated the clinical significance of M1 macrophage and CXCL9 as predictive biomarkers. We also explored the potential of developing a predictive model incorporating M1 macrophage, CXCL9, and RNA epigenetic modification‐related genes to stratify patients and guide precision immunotherapy.

## 2 RESULTS

### 2.1 Association of high M1 macrophage expression with improved survival and treatment response to ICIs

**FIGURE 1:**
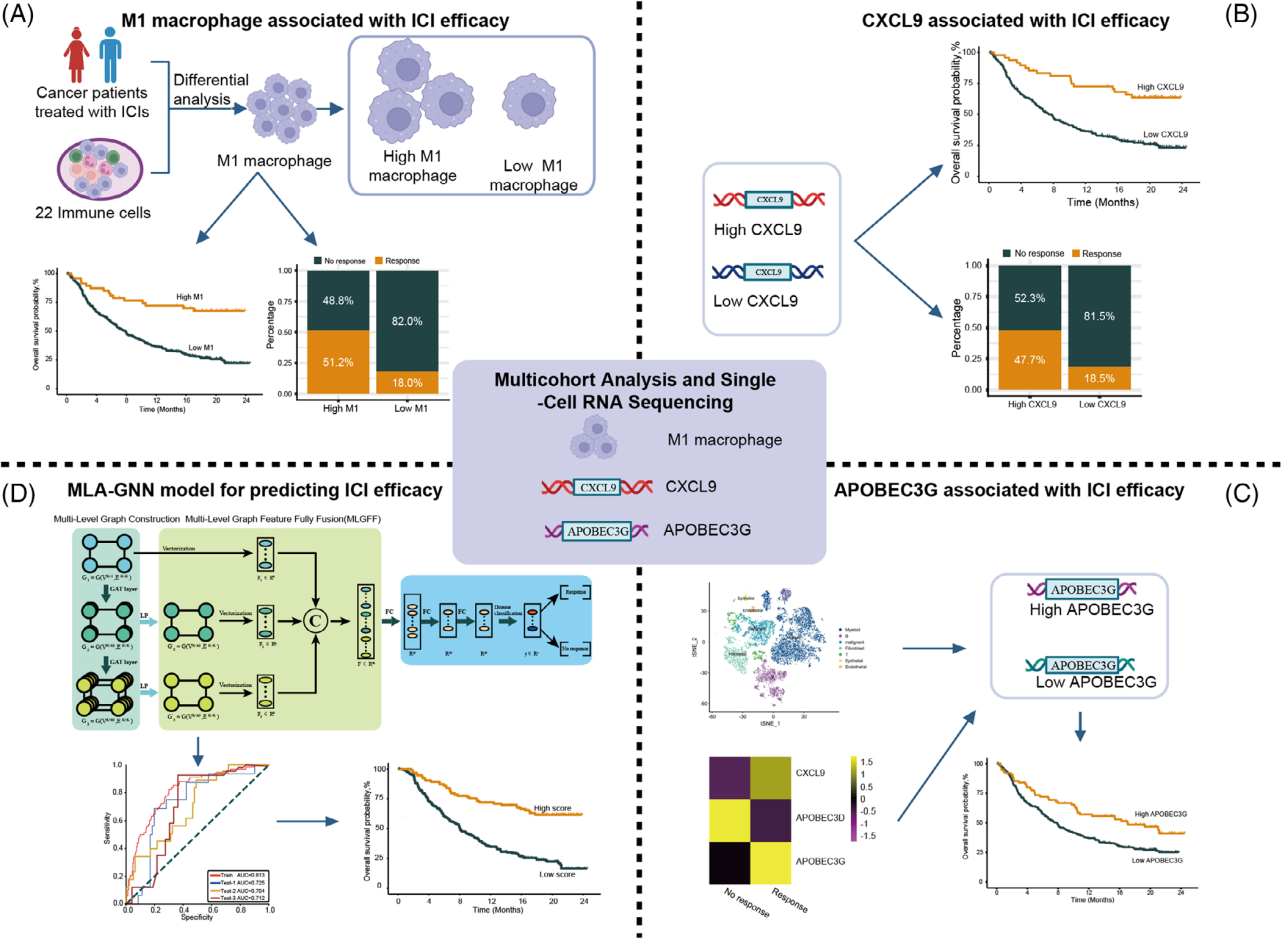
Study design overview. (A) A comprehensive approach was used to investigate the clinical significance and molecular characteristics of M1 macrophage in cancer patients treated with immune checkpoint inhibitors (ICIs). (B) The role of CXCL9, a chemokine involved in immune cell migration and activation in predicting ICI efficacy, was then investigated. The association between the expression levels of CXCL9 and clinical outcomes as well as immune checkpoint was evaluated. (C) To further understand the molecular mechanisms and interactions within the tumor immune microenvironment (TIME), the study performed combined analyses of transcriptomics and single‐cell RNA sequencing (scRNA‐seq) data. This approach aimed to identify novel biomarkers associated with ICI response, with a particular focus on the RNA epigenetic modification gene APOBEC3G. (D) Furthermore, a novel model was constructed using deep learning algorithms, specifically the multi‐level attention graph neural network (MLA‐GNN) model, based on M1 macrophage and TIME‐associated factors, to predict ICI efficacy.

**FIGURE 2:**
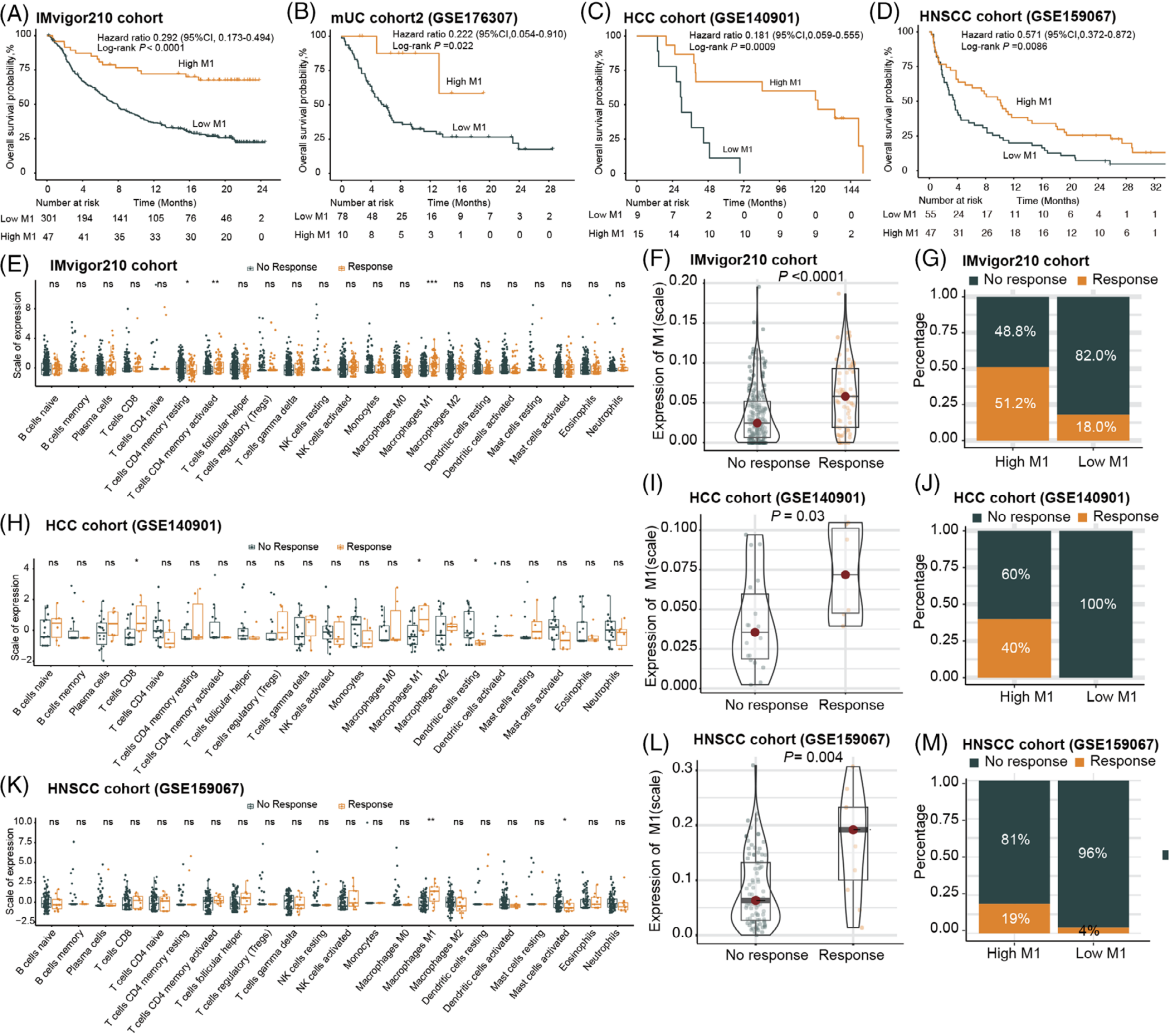
The expression levels of M1 macrophage are associated with favorable clinical outcomes in cancer patients undergoing treatment with immune checkpoint inhibitors (ICIs). (A–D) Kaplan–Meier curves illustrating the overall survival (OS) based on M1 macrophage infiltration in the IMvigor210 cohort (A), metastatic urothelial carcinoma (mUC) cohort2 (B), hepatocellular carcinoma (HCC) cohort (C), and head and neck squamous cell carcinomas (HNSCC) cohort (D). (E) CIBERSORT analysis of the IMvigor210 cohort, assessing the association between 22 immune cell types and the response to ICI therapy. (F and G) Association of M1 macrophage infiltration with the response to ICI therapy in the IMvigor210 cohort. (H) CIBERSORT analysis of the HCC cohort, investigating the association between 22 immune cell types and the response to ICI therapy. (I and J) Association of M1 macrophage infiltration with the response to ICI therapy in the HCC cohort. (K) CIBERSORT analysis of the HNSCC cohort, investigating the association between 22 immune cell types and the response to ICI therapy. (L and M) Association of M1 macrophage infiltration with the response to ICI therapy in the HNSCC cohort. In (F), (I), and (L), the significance of the difference was tested by Mann–Whitney test. In (E), (H) and (K), *p‐value ≤ 0.05, **p‐value ≤ 0.01, and ***p‐value ≤ 0.001.

Figure 1 presents a schematic representation of the study's design. We first employed a comprehensive approach to investigate the clinical significance and molecular characteristics of M1 macrophage in cancer patients treated with ICIs. In the IMvigor210 cohort, high expression of M1 macrophage showed a significant association with improved overall survival (OS) (*p* < 0.0001) (Figure 2A). Conversely, high expression of both M0 and M2 macrophages predicted worse OS (both *p*‐values < 0.001) (Figure S1A,B). Similarly, in the mUC cohort2, the hepatocellular carcinoma (HCC) cohort and the head and neck squamous cell carcinomas (HNSCC) cohort, high expression of M1 macrophage was remarkably associated with favorable OS (*p* = 0.022, 0.0009, and 0.0086, respectively) (Figure 2B‐D). Additionally, in patients from the Cancer Genome Atlas (TCGA) pan‐cancer cohort, high M1 macrophage infiltration was predictive of improved OS (Figure S2). After adjusting confounding variables, including age and stage, on the multivariate analysis for the mUC‐cohort2, high M1 macrophage expression remained an independent prognostic factor for improved OS (hazard ratio [HR] = 0.206, 95% confidence interval [CI] 0.050–0.854, *p* = 0.029) (Figure S3A). Additionally, in the HNSCC cohort, M1 macrophage retained its independent prognostic significance for OS after adjusting for age and gender (HR = 0.569, 95% CI 0.372–0.871, *p* = 0.009) (Figure S3B).

In the IMvigor210 cohort, the expression level of M1 macrophage was notably higher in the group responding to ICIs compared to the group that did not respond (*p* < 0.001) (Figure 2E,F). The high expression group of M1 macrophage exhibited a significantly higher objective response rate (ORR) compared to the low expression group (51.2% vs. 18.0%) (Figure 2G). The association between high M1 macrophage expression and improved ORR was further validated in the HCC cohort and the HNSCC cohort (Figure 2H–M).

### 2.2 Associations of M1 macrophage with immune phenotypes and checkpoints in the tumor microenvironment

**FIGURE 3:**
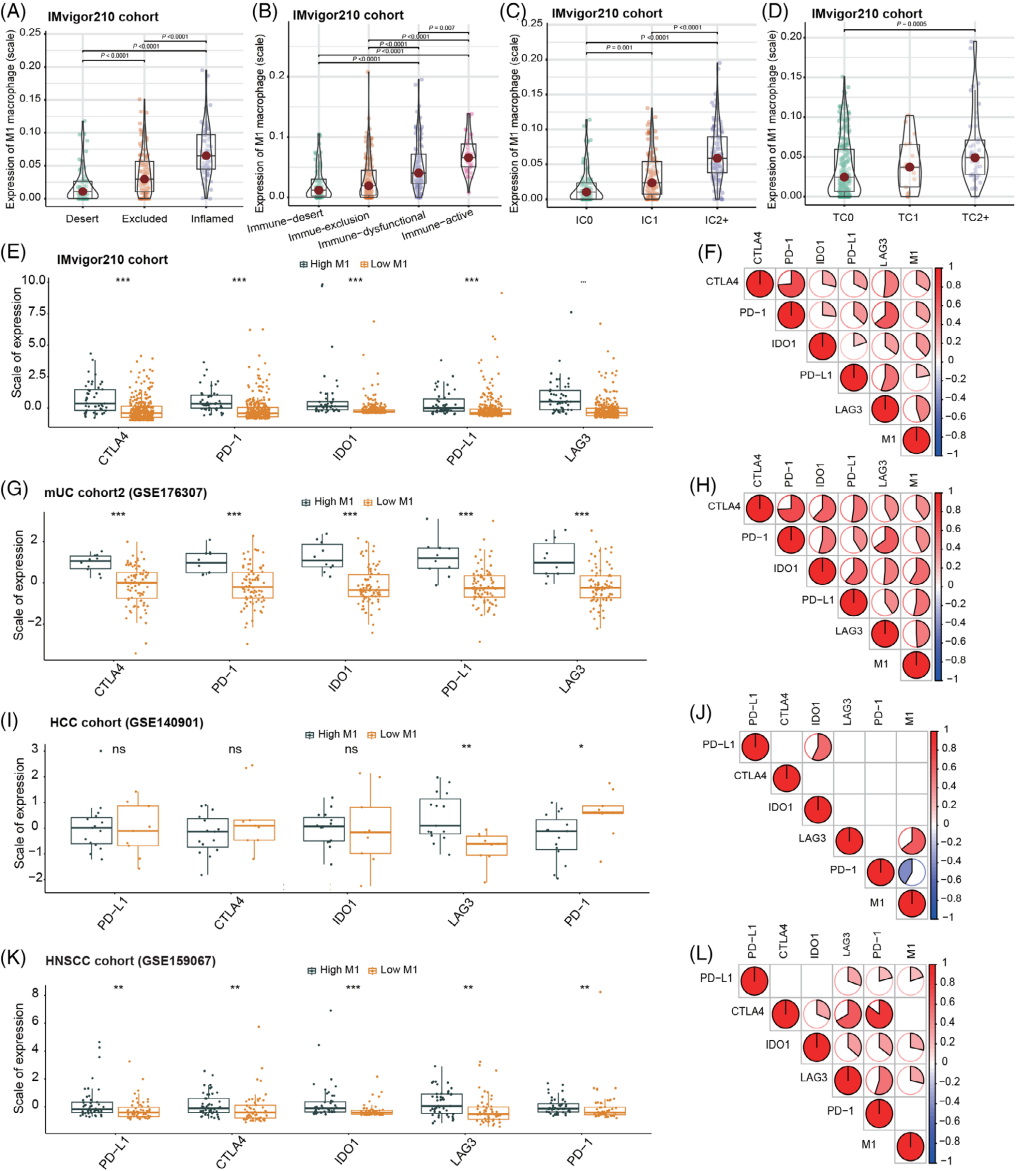
The expression levels of M1 macrophage are correlated with immune phenotypes and immune checkpoints. (A and B) The expression levels of M1 macrophage were evaluated in previously defined three immune phenotypes (A) and in the four immune phenotypes (B) based on the IMvigor210 cohort. (C and D) The expression levels of M1 macrophage were examined across different PD‐L1 expression levels by immune cells (C) and tumor cells (D) in the IMvigor210 cohort. (E, G, I, and K) The differences in expression levels of immune checkpoints between the high and low M1 macrophage groups were assessed in the IMvigor210 cohort (E), metastatic urothelial carcinoma (mUC) cohort2 (G), hepatocellular carcinoma (HCC) cohort (I), and head and neck squamous cell carcinomas (HNSCC) cohort (K). (F, H, J, and L) Spearman correlation analyses were conducted to investigate the correlation between M1 macrophage and immune checkpoints in the IMvigor210 cohort (F), mUC cohort2 (H), HCC cohort (J), and HNSCC cohort (L). PD‐1, anti‐programmed cell death‐1; PD‐L1, programmed cell death‐ligand 1; CTLA4, cytotoxic T‐lymphocyte‐associated protein 4; IDO1, indoleamine 2,3‐dioxygenase, LAG3, lymphocyte‐activation gene 3; IC, immune cells; TC, tumor cells. In (A–D), the significance of the difference was tested by Kruskal–Wallis test. In (E), (G), (I), and (K), *p‐value ≤ 0.05, **p‐value ≤ 0.01, and ***p‐value ≤ 0.001. In (F), (H), (J), and (L), the proportion of the pie charts represents the correlation coefficients.

To further elucidate the role of M1 macrophage associated with the TIME, we assessed the associations between M1 macrophage and immune phenotypes as well as immune checkpoints in the IMvigor210 cohort. Based on the three previously defined immune phenotypes,^17^ we observed that the expression level of M1 macrophage peaked in the immune‐inflamed phenotype and was at its minimal in the immune‐desert phenotype (Figure 3A). We also examined the distribution of M1 macrophage in the four immune phenotypes proposed earlier, which were based on a combination of long noncoding RNAs and tumor‐specific cytotoxic T lymphocytes.^18^ Consistently, M1 macrophage expression was markedly elevated in the immune‐active phenotype relative to the immune‐dysfunctional, immune‐exclusion, and immune‐desert phenotypes (all *p* values < 0.05) (Figure 3B).

Furthermore, we found that the expression of M1 macrophage was higher in patients with elevated levels of PD‐L1 expression by either ICs or tumor cells (TC) (Figure 3C,D). In line with this, high expression of M1 macrophage coexisted with increased infiltrations of other immune checkpoints, including cytotoxic T lymphocyte‐associated protein 4 (CTLA4), PD‐1, indoleamine 2,3‐dioxygenase, and lymphocyte‐activation gene 3 (Figure 3E–L). The analysis of breast cancer patients from the Sun Yat‐sen Memorial Hospital of Sun Yat‐sen University (SYSMH‐BC cohort) also illustrated substantial correlation between high M1 macrophage and elevated immune checkpoint expression (Figure S4). These data indicated that M1 macrophage might contribute to an immune‐active contexture within the TIME.

### 2.3 Associations of CXCL9 expression with M1 macrophage infiltration and ICI efficacy

**FIGURE 4:**
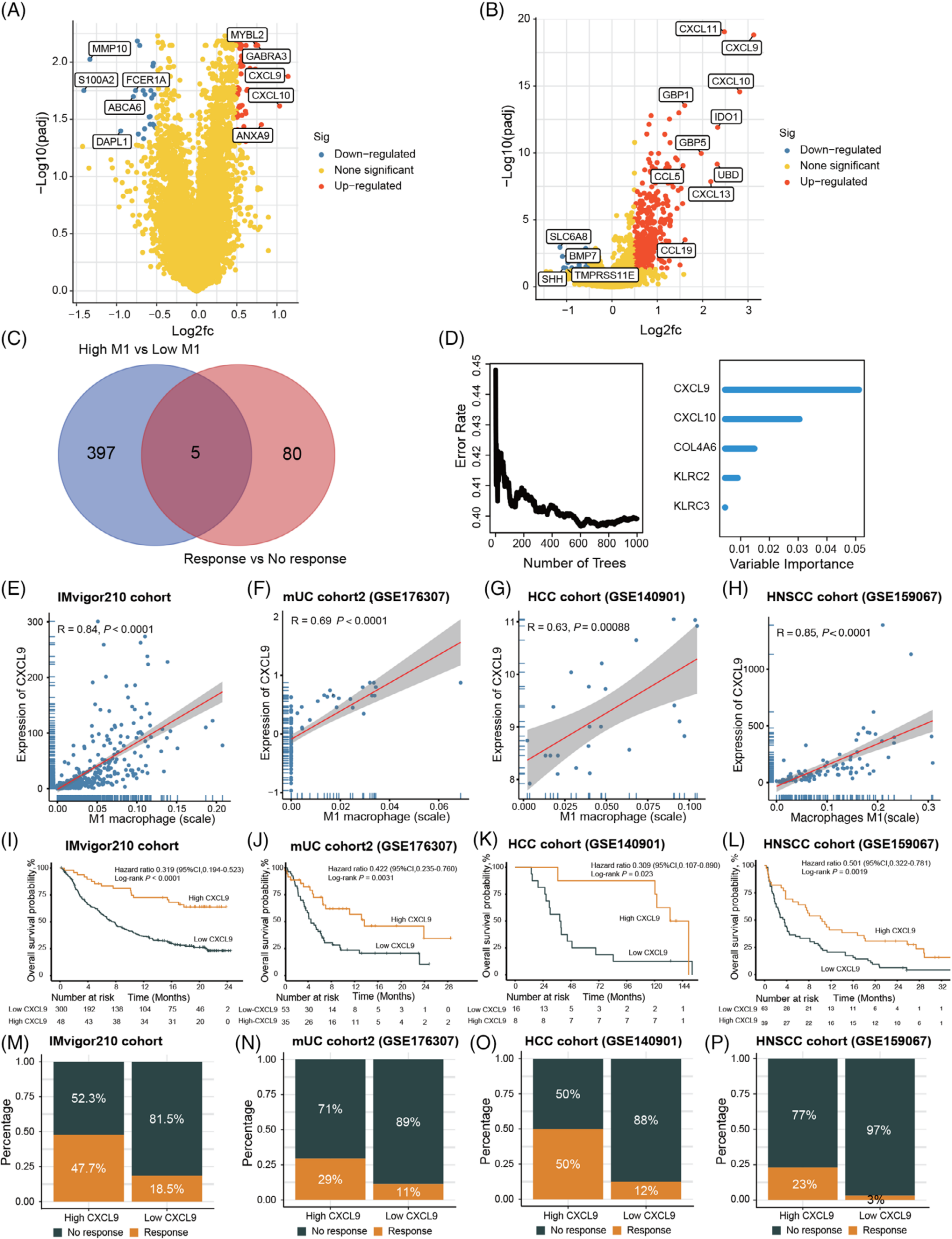
CXCL9 is closely associated with M1 macrophage and plays a significant role in influencing the clinical outcomes of immune checkpoint inhibitors (ICIs). (A) Volcano plot illustrating the differentially expressed genes (DEGs) between the ICI response and nonresponse groups based on the IMvigor210 cohort. (B) Volcano plot displaying the DEGs between the high and low M1 macrophage groups based on the IMvigor210 cohort. (C) Venn diagram depicting the intersection of the two sets of DEGs. (D) Random forest analysis highlighting CXCL9 as the most important feature. (E–H) Spearman correlation analysis demonstrating the correlation between M1 macrophage and CXCL9 expression in the IMvigor210 cohort (E), metastatic urothelial carcinoma (mUC) cohort2 (F), hepatocellular carcinoma (HCC) cohort (G), and head and neck squamous cell carcinomas (HNSCC) cohort (H). (I–L) Kaplan–Meier curves illustrating the overall survival (OS) according to CXCL9 expression in the IMvigor210 cohort (I), mUC cohort2 (J), HCC cohort (K), and HNSCC cohort (L). (M–P) Evaluation of the difference in objective response rate (ORR) between the high and low CXCL9 expression groups in the IMvigor210 cohort (M), mUC cohort2 (N), HCC cohort (O), and HNSCC cohort (P).

The IMvigor210 cohort was further analyzed to investigate the key marker genes that exhibited a high correlation with M1 macrophage infiltration and response to ICIs. Initially, we identified 85 genes differentially expressed between the ICI response and nonresponse groups, and additionally, 402 genes exhibited distinct expression patterns between the high and low M1 macrophage groups (Figure 4A,B). The protein–protein interaction (PPI) analysis revealed potential collaborative effects among proteins targeted by the top differentially expressed genes (DEGs) in the high and low M1 macrophage groups (Figure S5A). Additionally, based on the GENEMANIA database, the top DEGs exhibited interactions with 20 potential target genes (Figure S5B). Specifically, CXCL11, CXCL9, CXCL10, CXCL13, CCL19, and CCL5 were predominantly related to the cytokine‐ and chemokine‐related pathways, while GBP1 and GBP5 were involved in the response to interferon‐gamma pathway (Figure S5B). Notably, five genes (CXCL9, CXCL10, COLA4A6, KLRC2, and KLRC3) overlapped between the aforementioned two sets of DEGs (Figure 4C). Subsequently, these five genes underwent further selection using random forest analysis, which revealed CXCL9 as the most important gene for subsequent analyses (Figure 4D). Correlation analyses consistently demonstrated a positive correlation between CXCL9 expression and M1 macrophage in the IMvigor210 cohort (*R* = 0.84, *p *< 0.0001), mUC‐cohort2 (*R* = 0.69, *p *< 0.0001), HCC cohort (*R* = 0.63, *p *= 0.0009), and HNSCC cohort (*R* = 0.85, *p *< 0.0001) (Figure 4E–H).

In the IMvigor210 cohort, high expression of CXCL9 was significantly associated with superior OS (*p *< 0.0001) (Figure 4I) and a higher ORR (47.7% vs. 18.5%) (Figure 4M, Figure S6A). This association was further validated in the mUC cohort2 (OS: *p *= 0.0031; ORR: 29% vs. 11%), HCC cohort (OS: *p *= 0.023; ORR: 50% vs. 12%), and HNSCC cohort (OS: *p *= 0.0019; ORR: 23% vs. 3%) (Figure 4J–L, N–P; Figure S6B–D). Moreover, high CXCL9 expression predicted better OS in the TCGA pan‐cancer cohort (*p *< 0.001) (Figure S7).

### 2.4 Impact of CXCL9 on the TIME and its correlation with immune phenotypes and checkpoints

To gain insights into the functional role of CXCL9, a chemokine involved in IC migration and activation, in predicting ICI efficacy, we further explored its potential influence on the TIME. Using data from the IMvigor210 cohort, we compared gene expression profiles between the high CXCL9 expression group and the low expression group, resulting in the identification of 1359 DEGs (Figure S8A). Gene ontology (GO) pathway enrichment analysis revealed that these DEGs were significantly related with immunologic processes, including T‐cell activation, MHC protein complex, and immune receptor activity (Figure S8B). Additionally, Kyoto Encyclopedia of Genes and Genomes (KEGG) enrichment analysis demonstrated enrichment in antigen processing and presentation, and cytokine–cytokine receptor interaction processes (Figure S8C).

**FIGURE 5:**
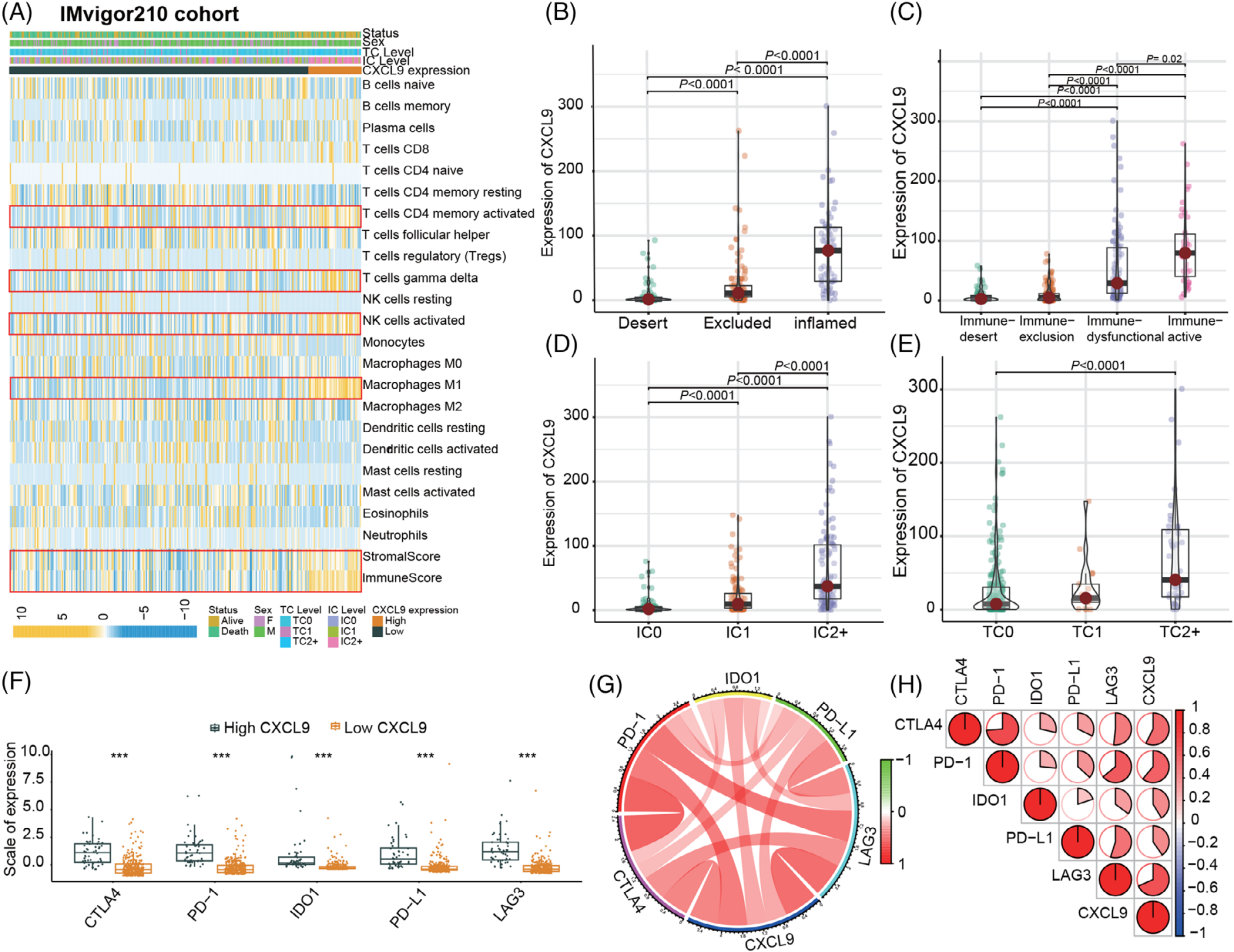
CXCL9 expression is significantly higher in the immune‐inflamed and immune‐active phenotypes based on the IMvigor210 cohort. (A) Heatmap representing the CIBERSORT analysis, which assesses the correlation between the expression level of CXCL9 and 22 different types of immune cells. (B and C) Expression levels of CXCL9 in the previously defined three immune phenotypes (B) and the four immune phenotypes (C). (D and E) Expression levels of CXCL9 across different PD‐L1 expression levels by immune cells (D) and tumor cells (E). (F) Difference in expression levels of immune checkpoints between the high and low CXCL9 expression groups. (G and H) Spearman correlation analysis investigating the correlation between CXCL9 and immune checkpoints. GO, gene ontology; KEGG, Kyoto Encyclopedia of Genes and Genomes; DEGs, differentially expressed genes; PD‐1, anti‐programmed cell death‐1; PD‐L1, programmed cell death‐ligand 1; CTLA4, cytotoxic T‐lymphocyte‐associated protein 4; IDO1, indoleamine 2,3‐dioxygenase, LAG3, lymphocyte‐activation gene 3; IC, immune cells; TC, tumor cells. In (B–E), the significance of the difference was tested by Kruskal–Wallis test. In (F), ***p‐value ≤ 0.001.

Further analyses using the CIBERSORT algorithm indicated that patients with high CXCL9 expression exhibited elevated levels of active CD4^+^ T cells, T‐cell gamma delta, active natural killer (NK) cells, and M1 macrophage (Figure 5A, Figure S9). Moreover, high CXCL9 expression positively correlated with StromalScore and ImmuneScore, indicating a more pronounced stromal and IC infiltration in the TIME (Figure 5A).

We investigated the correlations between CXCL9 expression and immune phenotypes as well as immune checkpoints based on the IMvigor210 cohort. Consistently, CXCL9 expression was significantly higher in the immune‐inflamed and immune‐active phenotypes (Figure 5B,C). Additionally, CXCL9 expression positively correlated with PD‐L1 expression (by either IC or TC) (Figure 5D,E) and other immune checkpoints (Figure 5F–H). These positive associations between CXCL9 expression and immune checkpoints were also observed in the mUC cohort2, HCC cohort, HNSCC cohort, and SYSMH‐BC cohort (Figure S10A–I). Collectively, these findings demonstrated that high CXCL9 expression was indicative of a tumor immune‐active microenvironment, which might enhance the sensitivity of tumors to ICIs.

### 2.5 RNA epigenetic modifications and APOBEC3G: Implications for antitumor immune response and prognosis

Previous evidence suggests that RNA epigenetic modifications play a crucial role in regulating dynamic macrophage polarization, thereby influencing cancer growth and metastasis.^16^, ^19^ To further understand the molecular mechanisms and interactions within the TIME, the study performed combined analyses of transcriptomics and scRNA‐seq data. This approach aimed to identify novel biomarkers associated with ICI response, with a particular focus on RNA modification genes. Among the 1357 DEGs identified between the CXCL9 high expression group and the low expression group, two genes, namely, APOBEC3D and APOBEC3G, were found to overlap within the 184 RNA modification genes associated with the “GOBP RNA MODIFICATION” pathway in the Molecular Signatures Database.

**FIGURE 6:**
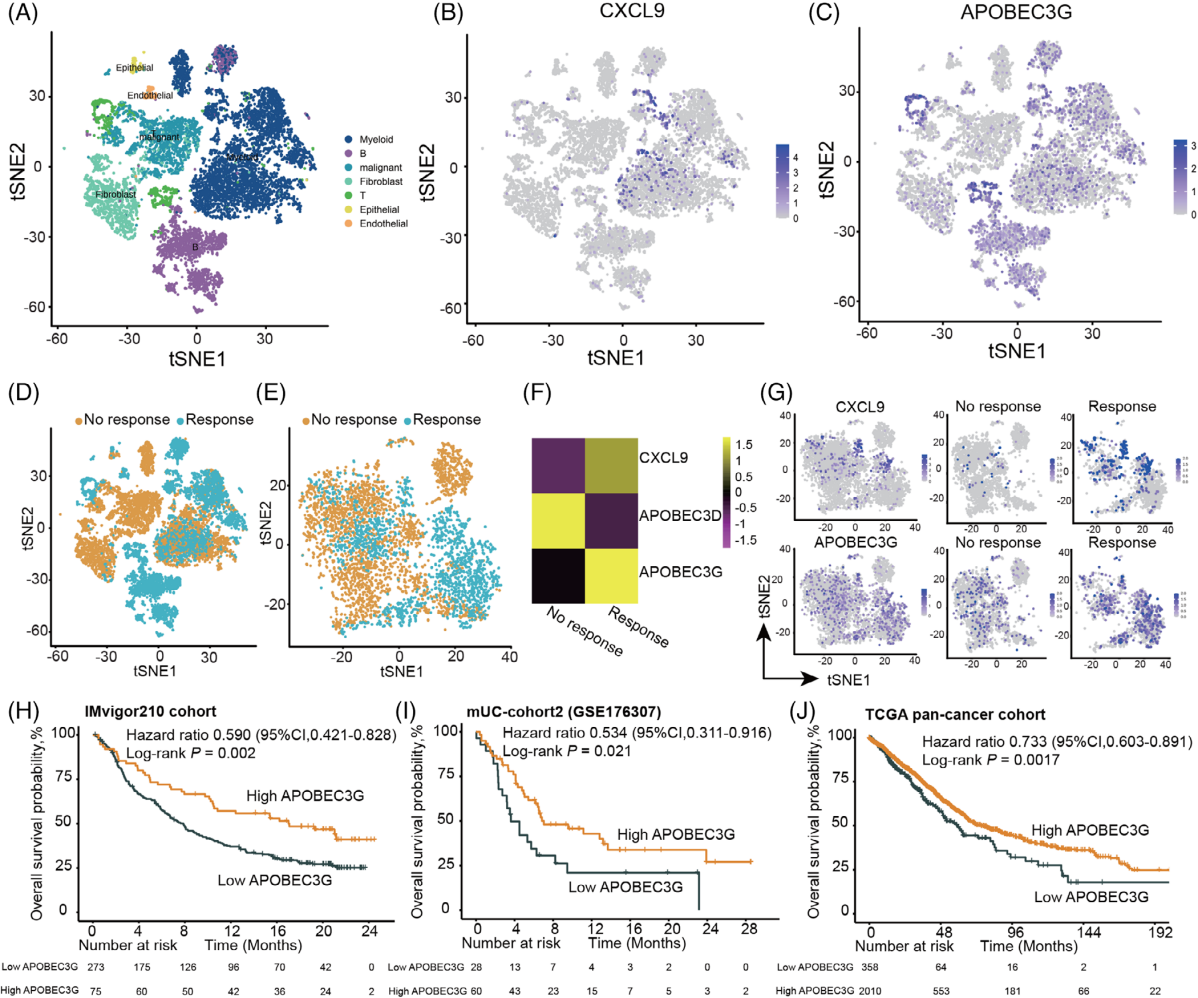
APOBEC3G is identified to be positively correlated with the response to immune checkpoint inhibitors (ICIs) and the tumor immune microenvironment (TIME). (A) Distribution of the seven cell clusters visualized by t‐distributed stochastic neighbor embedding (t‐SNE), colored according to the respective cell clusters. (B) t‐SNE plot displaying the expression levels of CXCL9. (C) t‐SNE plot illustrating the expression levels of APOBEC3G. (D) t‐SNE plot showing two triple negative breast cancer (TNBC) samples stratified based on their response to ICI therapy, with one classified as a responder and the other as a nonresponder. (E) t‐SNE plot highlighting the distinction between responders and nonresponders within the macrophage cluster. (F) Heatmap depicting the expression levels of CXCL9, APOBEC3D, and APOBEC3G between responders and nonresponders within the macrophage cluster. (G) t‐SNE plot demonstrating the expression levels of CXCL9 and APOBEC3G within the macrophage cluster. (H–J) Kaplan–Meier curves depicting the overall survival (OS) based on the expression of APOBEC3G in the IMvigor210 cohort (H), metastatic urothelial carcinoma (mUC) cohort2 (I), and TCGA‐Pancancer cohort (J).

To further explore the significance of these genes, we performed scRNA‐seq using fresh tumor samples from two triple negative breast cancer (TNBC) patients who received anti‐PD‐1 antibody‐based combinational treatment. Through *t*‐distributed stochastic neighbor embedding (t‐SNE) visualization, we identified seven distinct cell clusters based on lineage‐specific genes (Figure 6A, Figure S11). Notably, CXCL9 was predominantly expressed in myeloid clusters, while APOBEC3G exhibited a more widespread distribution across myeloid, B‐lymphocyte, and T‐lymphocyte clusters (Figure 6B,C). Subsequently, we specifically identified the macrophage cluster within the myeloid clusters (Figure 6D,E). Within the macrophage cluster, both CXCL9 and APOBEC3G showed a positive correlation with response to ICI therapy, whereas APOBEC3D exhibited a contrasting pattern (Figure 6F). Responders demonstrated higher expression levels of CXCL9 and APOBEC3G (Figure 6G). Based on these findings, APOBEC3G was selected for further analysis.

Further analysis of transcriptomic data from the IMvigor210 cohort revealed a moderate correlation between APOBEC3G expression and CXCL9 expression as well as M1 macrophage infiltration (Figure S12A,B). APOBEC3G also displayed a moderate correlation with CD4^+^ T‐cell infiltration (Figure S12C). Functional analysis based on DEGs between the high and low APOBEC3G expression groups suggested that APOBEC3G may be involved in immune‐related pathways, including T‐cell activation, immune receptor activity, and antigen processing and presentation (Figure S13). Moreover, high APOBEC3G expression was significantly associated with better OS in the IMvigor210 cohort, mUC cohort2, and TCGA pan‐cancer cohort (Figure 6H–J).

### 2.6 Construction of a novel model for predicting clinical outcomes of ICIs based on M1 macrophage, CXCL9, and APOBEC3G

**FIGURE 7:**
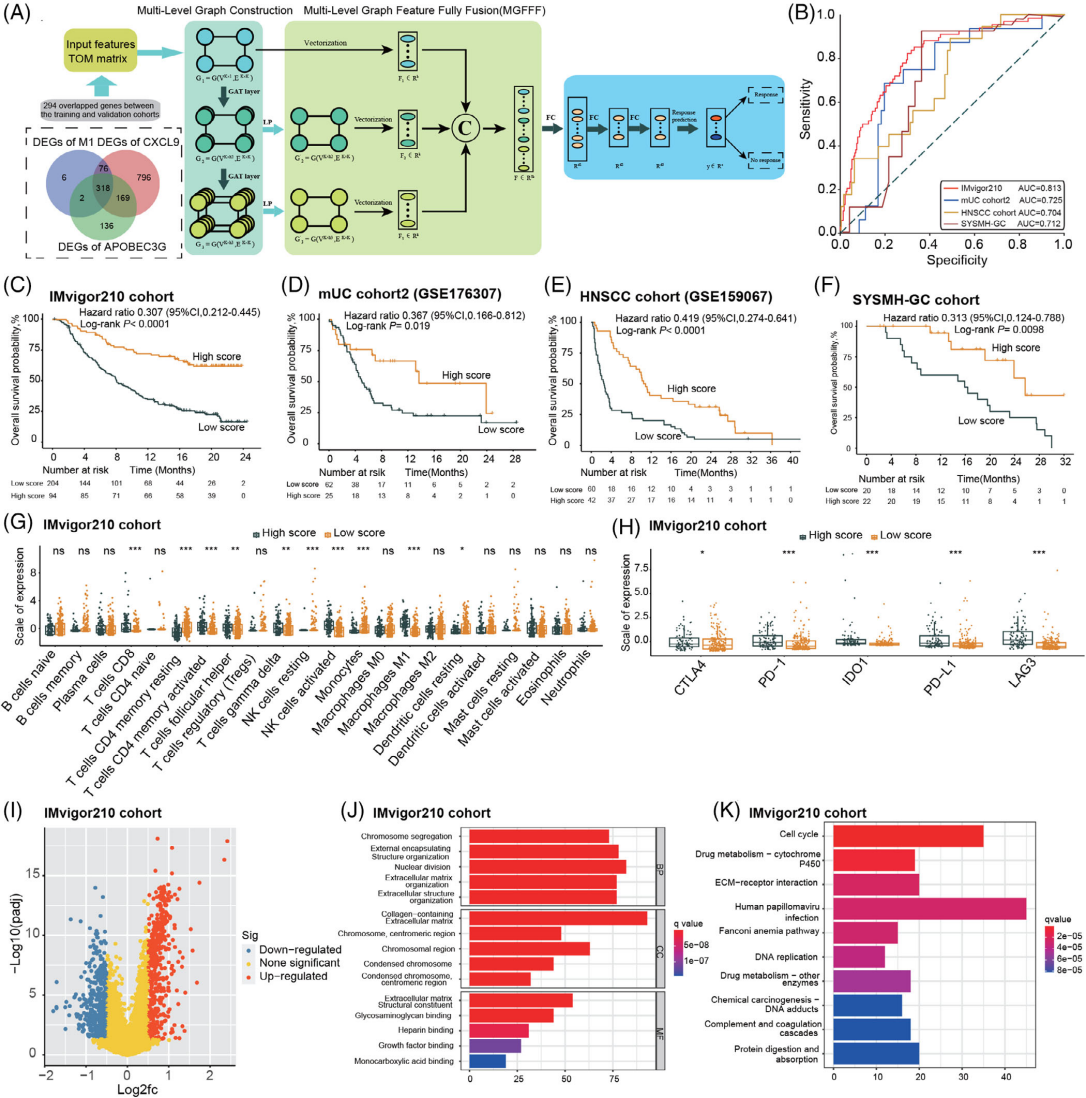
A novel model was constructed for predicting the outcomes of immune checkpoint inhibitors (ICIs) based on M1 macrophage, CXCL9, and APOBEC3G‐related genes. (A) Overview of the multi‐level attention graph neural network (MLA‐GNN) model. (B) Evaluation of the performance of the novel model in predicting the objective response rate (ORR) of ICIs in the IMvigor210 cohort, the metastatic urothelial carcinoma (mUC) cohort2, the head and neck squamous cell carcinomas (HNSCC) cohort, and the Sun Yat‐sen Memorial Hospital‐gastric cancer (SYSMH‐GC) cohort. (C–F) Kaplan–Meier curves illustrating the overall survival (OS) based on the novel model score in the IMvigor210 cohort (C), the mUC cohort2 (D), the HNSCC cohort (E), and the SYSMH‐GC cohort (F). (G) CIBERSORT analysis of the IMvigor210 cohort, assessing the association between the novel model score and 22 different types of immune cells. (H) Comparison of the expression levels of immune checkpoints between the high score and low score groups in the IMvigor210 cohort. (I) Volcano plot illustrating the differentially expressed genes (DEGs) between the high score and low score groups in the IMvigor210 cohort. (J–K) Gene ontology (GO) (J) and Kyoto Encyclopedia of Genes and Genomes (KEGG) (K) pathway enrichment analyses conducted on the DEGs. PD‐1, anti‐programmed cell death‐1; PD‐L1, programmed cell death‐ligand 1; CTLA4, cytotoxic T‐lymphocyte‐associated protein 4; IDO1, indoleamine 2,3‐dioxygenase, LAG3, lymphocyte‐activation gene 3. In (A), the first step involved examining the intersection of three sets of DEGs from the M1 macrophage, CXCL9, and APOBEC3G high and low groups in the IMvigor210 cohort. The genes that overlapped among these sets were used to construct the topological overlap matrix. Next, multi‐level graphs were built using the Multi‐Level Graph Construction module, which utilized graph attention layers. Following that, local gene‐level features and global pathway‐level features were fused after linear projection and vectorization, utilizing the Multi‐level Graph Feature Fully Fusion module. Finally, the fused feature was passed through the Multi‐Task Prediction module to perform the prediction of ICI response. In (B), the area under curve (AUC) values of 0.813 and 0.725 were obtained based on all patients from the IMvigor210 cohort (*n* = 348) and the mUC‐cohort2 (*n* = 88), respectively. In (E and F), *p‐value ≤ 0.05, **p‐value ≤ 0.01, and ***p‐value ≤ 0.001.

Furthermore, we aimed to conduct a novel model using deep learning algorithms, specifically the multi‐level attention graph neural network (MLA‐GNN) model. Given the consistent association of high M1 macrophage, CXCL9, and APOBEC3G expression with improved outcomes of ICIs, genes associated with these factors were utilized to develop a novel model for predicting clinical outcomes of ICIs. Among the three sets of DEGs in the IMvigor210 cohort, a total of 318 genes overlapped. Among these, 294 genes were shared between the IMvigor210 cohort and mUC cohort2, and were subsequently used to construct the novel model using MLA‐GNN. An overview of the MLA‐GNN is presented in Figure 7A. The novel model demonstrated favorable predictive ability for ORR in patients receiving ICIs, with area under curve (AUC) values of 0.813, 0.725, 0.704, and 0.712 for the IMvigor210 cohort, mUC cohort2, HNSCC cohort, and SYSMH‐gastric cancer (GC) cohort, respectively (Figure 7B).

The predictive performance of the novel model was compared with that of PD‐L1 and tumor mutation burden (TMB) based on patient who had data on PD‐L1 and/or TMB. In the IMvigor210 cohort, the novel model achieved an AUC of 0.780, outperforming PD‐L1 (AUC = 0.560) and TMB (AUC = 0.720) (Figure S14A). Similarly, in the mUC cohort2, the AUC of the novel model (0.730) for predicting ORR was higher than that of PD‐L1 (AUC = 0.620) (Figure S14B). Moreover, utilizing the scores from the newly developed model, patients were segregated into groups with high scores and low scores. Notably, patients in the high‐score category exhibited a markedly improved OS compared to their low‐score counterparts in the IMvigor210 cohort, mUC cohort2, HNSCC cohort, and SYSMH‐GC cohort (Figure 7C–F).

In comparison to those with low scores, individuals in the high‐score group showed increased expression levels of diverse IC types, including CD8^+^ T cells, active CD4^+^ T cells, T‐cell gamma delta, active NK cells, and M1 macrophage (Figure 7G). Moreover, patients with high scores showed elevated expression levels of immune checkpoints compared to those with low scores (Figure 7H). Pathway enrichment analyses, based on the DEGs between the high‐score and low‐score groups, revealed that the novel model was highly enriched in pathways associated with the extracellular matrix, cell cycle, DNA replication, and drug metabolism (Figure 7I–K).

## 3 DISCUSSION

This study provides a comprehensive investigation into the role of M1 macrophage and the molecular mechanisms associated with the TIME in determining the response to ICIs in cancer patients. Moreover, the associations of CXCL9 and APOBEC3G with ICI responses further support their relevance as valuable prognostic markers. In addition to the molecular insights gained, we present a novel predictive model for ICI response based on M1 macrophage and TIME‐associated factors, utilizing the MLA‐GNN model. This model demonstrates superior predictive performance compared to conventional markers such as PD‐L1 expression or TMB.

The consistent association between high expression of M1 macrophage and improved OS as well as treatment response, as observed across different cohorts, underscores the prognostic and predictive significance of M1 macrophage. Our results align with previous studies that have demonstrated the favorable role of M1 macrophage in promoting antitumor immune responses and improving patient outcomes.^20^ It has been reported that anti‐PD‐L1 therapy can induce remodeling of the macrophage compartment in responsive tumor models, primarily through increased levels of IFNγ, leading to a more proinflammatory phenotype and enhanced activity of CD8 T cells.^21^ Notably, the presence of macrophages exhibiting a dual M1‐like and M2‐like phenotype, referred to as M1hot, has been associated with robust T‐cell responses and improved survival outcomes in lung cancer.^15^ These findings are consistent with prior studies that have highlighted the favorable role of M1 macrophage in promoting antitumor immune responses and improving patient outcomes. The pro‐inflammatory and tumoricidal functions of M1 macrophage make them a crucial component of an effective immune response against tumors. Conversely, high expression of M0 and M2 macrophages, which are associated with immunosuppressive and protumoral effects, predicts worse OS. This highlights the importance of discerning macrophage subtypes and their functional states when evaluating the landscape of the TIME. Additionally, these insights underscore the potential therapeutic relevance of developing strategies that modulate the balance between M1 and M2 macrophages to optimize the efficacy of immunotherapy, thereby enhancing the overall clinical outcomes for cancer patients.

The associations between M1 macrophage and immune phenotypes provide valuable insights into the immune contexture of the tumor microenvironment (TME). The higher expression of M1 macrophage in the immune‐inflamed and immune‐active phenotypes suggests their involvement in an immune‐responsive environment. This aligns with previous studies demonstrating that an inflamed TME characterized by IC infiltration is associated with improved responses to ICIs. The coexistence of M1 macrophage with increased infiltrations of immune checkpoints indicates a potential interplay between M1 macrophage and immune checkpoint pathways. These observations suggest that M1 macrophage may contribute to an immunologically active contexture within the TIME, enhancing the sensitivity of tumors to ICIs. Overall, the identification of M1 macrophage as a key player in the contexture of TIME underscores its significance as a potential target for therapeutic interventions. Furthermore, the observation of the interplay between M1 macrophage and immune checkpoint pathways offers promising prospects for the development of combination therapies that target both M1 macrophages and specific immune checkpoints, thereby fostering a more effective anti‐tumor immune response.

The efficacy of ICI therapy largely relies on the recruitment of tumor‐infiltrating lymphocytes (TILs). However, the precise mechanisms underlying TIL recruitment in the context of ICIs are not yet fully understood. The CXCR3 chemokine and its ligands, including CXCL9, CXCL10, and CXCL11, play crucial roles in the migration, differentiation, and activation of ICs.^22^ CXCL9, also known as monokine induced by IFN‐γ, is primarily responsible for inducing lymphocytic infiltration into tumors, leading to the suppression of tumor growth.^23^ In the context of PD‐1 blockade, the CXCR3 chemokine system has been proposed to be essential for the functional CD8^+^ T‐cell response, with CXCL9 derived from CD103^+^ dendritic cells playing a pivotal role in this process. Moreover, CXCL9 and CXCL10 have been identified as potential early biomarkers for predicting the response to ICI.^10^ High expression of CXCL9 and CXCL10 has been strongly associated with improved survival and a substantial increase in the number of intratumoral CD8^+^ T cells in cancer patients treated with ICIs. Interestingly, a study has demonstrated that macrophages are also a major source of CXCL9, both in preclinical models and in clinical patients, and this production is dependent on IFN‐γ.^9^

In this study, the strong correlation between high CXCL9 expression and M1 macrophage infiltration further underscores the role of CXCL9 as a valuable biomarker in ICI therapy. CXCL9 functions as a chemoattractant for effector T cells, integral to the immune responses against tumors. The positive association between high CXCL9 expression and superior OS as well as higher response rates to ICIs emphasizes the importance of CXCL9‐mediated immune activation in determining treatment outcomes. Moreover, the enrichment of immune‐related pathways, and the correlation between CXCL9 expression and immune checkpoints support the notion that CXCL9 contributes to an immune‐active microenvironment. Taken together, the robust association between CXCL9 and enhanced treatment responses to ICIs illuminates a potential avenue for patient stratification and personalized therapeutic interventions. Elevated CXCL9 expression can serve as a reliable biomarker to identify patients with a higher likelihood of benefiting from ICI therapy. Moreover, these findings underscore the potential for developing therapeutic strategies which modulate CXCL9 expression to enhance the clinical impact of ICIs.

Furthermore, through comprehensive analyses of transcriptomics and scRNA‐seq data, this study identifies APOBEC3G, an RNA modification gene, as significantly associated with the survival benefit derived from ICIs. This highlights the potential role of RNA modification processes in shaping the response to ICIs. APOBEC3, a member of the large apolipoprotein B mRNA editing enzyme catalytic polypeptide‐like family, plays a critical role in innate immunity by inducing mutations in viral DNA and restricting viral replication.^24^ The APOBEC3 family is highly expressed in macrophages, lymphoid cells, and dendritic cells, and dysregulated APOBEC3 activity has been implicated in genome mutagenesis in cancer.^24^, ^25^, ^26^ Notably, high expression of APOBEC3G, one of the APOBEC3 genes, has shown a strong correlation with T‐cell infiltration and improved outcomes in patients with high‐grade serous ovarian carcinoma.^27^ APOBEC3G also exhibited significant positive correlations with PD‐L1, suggesting that APOBEC‐driven mutagenesis may contribute to an active immune microenvironment in estrogen receptor‐positive breast cancer.^28^ However, contrasting findings have been reported in kidney renal clear cell carcinoma, where APOBEC3G positively correlated with the expression of immune checkpoints and immunosuppressive cells, serving as a potential biomarker for poor prognosis.^29^ Despite the observed association between APOBEC3G and immune infiltration, it remains unclear whether APOBEC3G can influence the clinical outcomes of ICIs.

Our finding that APOBEC3G was associated with ICI response and improved OS highlights the potential influence of RNA epigenetic modifications on the antitumor immune response. The positive correlation between APOBEC3G expression and response to ICI therapy further supports the role of APOBEC3G as a prognostic marker. This finding prompts the consideration of APOBEC3G as a potential tool for patient stratification, enabling clinicians to accurately predict patient clinical outcomes to ICI treatment and tailor therapy regimens accordingly. Interestingly, the results of our scRNA‐seq analysis indicated that high expression of APOBEC3G within macrophages was associated with benefits from ICI therapy. This observation suggests the potential involvement of APOBEC3G in the regulation of macrophage‐mediated immune responses and underscores the significance of macrophage‐associated mechanisms in enhancing the efficacy of ICIs. Furthermore, APOBEC3G was correlated with immune‐related pathways. Despite these data, the important influence of APOBEC3G and RNA epigenetic modifications on ICI efficacy and detailed mechanisms underlying this need further elucidation.

In addition, we developed a novel model incorporating M1 macrophage, CXCL9, and APOBEC3G‐relatde genes. This innovative model offers a promising tool for predicting the clinical outcomes of ICIs, which can substantially enhance patient care and treatment decision‐marking. This model exhibited superior predictive ability compared to conventional markers such as PD‐L1 and TMB, emphasizing the importance of considering the comprehensive immune landscape in treatment response prediction. The increased expression of IC types and immune checkpoints in the high‐score group suggests a more favorable TIME for ICI efficacy. These findings support the potential clinical utility of the novel model in identifying patients who are most likely to benefit from ICI therapy, thus optimizing treatment outcomes and minimizing unnecessary exposure to potential side effects in patients who are unlikely to respond.

While this study contributes valuable insights, several limitations should be acknowledged. First, the analyses were primarily based on retrospective cohorts, and prospective validation is required to confirm the findings. Since the study encompassed several study cohorts involving ICI therapy, there may be heterogeneity in patients and treatment across study cohorts. Caution should be paid when interpreting the results. Second, the sample size of the GSE140901 cohort is relatively small (*n* = 24), which can probably explain the nonstatistically significant association between M1 macrophage or CXCL9 and several immune checkpoints in this cohort. Additionally, the direct or indirect interactions involving APOBEC3G, CXCL9, or M1 macrophages were not investigated in this study. More studies delving into the molecular pathways and interactions between M1 macrophage, CXCL9, and APOBEC3G within the TME may provide further insights into the underlying biology. Future investigations with cell or animal experiments are necessary to elucidate the functional mechanisms underpinning these observed associations.

## 4 CONCLUSIONS

In conclusion, the findings highlight the clinical significance of M1 macrophage and CXCL9 as potential biomarkers for patient stratification and indicate the potential importance of RNA modification processes in impacting cancer immunotherapy response. The novel predictive model based on M1 macrophage and TIME‐associated factors offers promise for improving patient selection and facilitating the implementation of cancer precision immunotherapy strategies. Further research is warranted to elucidate the underlying mechanisms and validate the findings in prospective clinical trials.

## 5 METHODS AND MATERIALS

### 5.1 Patients and study design

The study incorporated patients from various sources, resulting in the inclusion of the following patient cohorts: First, a total of 348 patients with metastatic mUC who received treatment with the PD‐L1 inhibitor atezolizumab were included from the single‐arm, phase 2, multicenter IMvigor210 trial.^30^ Second, another cohort consisted of 88 patients with mUC who underwent anti‐PD1/PD‐L1 therapies and were part of the mUC cohort2. These patients were sourced from the GEO dataset GSE176307.^31^ Additionally, 24 patients with advanced/metastatic HCC who were treated with ICIs were included as the HCC cohort. These patients were obtained from the GEO dataset GSE140901.^32^ Furthermore, 102 patients with advanced HNSCC who underwent PD‐1/PD‐L1 targeted immunotherapy from the GSE159067 cohort were included as the HNSCC cohort.^33^ The transcriptomic and corresponding clinical data from the IMvigor210 trial were obtained using the R package IMvigor 210 Core Biologies.^30^ For the GEO datasets, transcriptomic and related clinical data were retrieved from the GEO database (http://www.ncbi.nlm.nih.gov/geo/). The patients lacking survival data were omitted from the analyses. Detailed characteristics of patients are shown in Table S1.

Moreover, the analysis encompassed a cohort of 99 breast cancer patients who underwent standard treatment. Among them, 97 patients had available RNA‐seq data from SYSMH‐BC cohort. scRNA‐seq was conducted on two TNBC patients who received PD‐1 antibody‐based combinational treatment at Sun Yat‐sen Memorial Hospital. Among the two patients, one exhibited a positive response to ICIs, while the other did not achieve a response. Additionally, 44 gastric cancer patients undergoing anti‐PD‐1 therapy with RNA‐seq data from Sun Yat‐sen Memorial Hospital (SYSMH‐GC cohort) were included for the subsequent model validation.

In addition, a pan‐cancer cohort, referred to as the Pancancer‐cohort, was incorporated from TCGA. The Pancancer‐cohort included the following patient numbers for each respective cancer type: 928 patients with breast cancer, 446 patients with colon adenocarcinoma, 88 patients with rectum adenocarcinoma, 495 patients with lung squamous cell carcinoma, 376 patients with ovarian serous cystadenocarcinoma, 459 patients with skin cutaneous melanoma, and 504 patients with thyroid carcinoma.

The study focused on two primary endpoints: OS and ORR. Patients were divided into two groups based on their response to ICIs: the response group, which included patients with complete response and partial response, and the non‐response group, which included patients with stable disease and progressive disease. The study adhered to the principles outlined in the Declaration of Helsinki and received approval from the ethics committee of Sun Yat‐sen Memorial Hospital, Sun Yat‐sen University (Approval Number: SYSEC‐KY‐KS‐2019‐171‐001). Due to the retrospective nature of the study and the utilization of publicly available datasets, the requirement for informed consent from study participants was waived by the ethics committee.

### 5.2 Quantification of IC proportions, DEG analyses, and identification of key marker genes

*Immune cell quantification*: The proportions of 22 types of ICs, including seven T‐cell types, naïve and memory B cells, plasma cells, NK cells, and myeloid subsets, were quantified using the CIBERSORT algorithm.^34^ This quantification was performed with the LM22 signature matrix and a 100× permutation count, without applying quantile normalization. CIBERSORT was utilized to analyze the relative expression levels of 547 genes in each tissue samples utilizing the high‐dimensional genomic data derived from bulk tissue samples. Consequently, the normalized gene expression profiles were converted into the proportion of 22 tumor‐infiltrating ICs. Macrophages consisted of M0, M1, and M2 subtypes. Heatmaps were generated to visualize the differences in IC expression levels among different groups.

*DEG analyses*: All DEG analyses were performed using the R package “Limma.”^35^ DEGs were identified using the Wilcoxon test, considering absolute log2 (fold change) values > 0.5 and *p* value < 0.05 as criteria for significance. Volcano plots were generated to display the DEGs between two groups.

*GO and KEGG pathway enrichment analyses*: The R package “clusterProfiler”^36^ was utilized for conducting GO and KEGG pathway enrichment analyses. Biological functions encompassing cellular component, molecular function, and biological process were identified using Fisher's exact test, with false discovery rate‐corrected *p* value < 0.05 considered statistically significant.

*Identification of key marker genes*: The process to pinpoint key marker genes linked to M1 macrophage infiltration and ICI response began with analyzing DEGs between groups with high and low M1 macrophage expression, and between the response and non‐response groups in the IMvigor210 cohort. Subsequently, the overlapping genes among these DEGs were identified. Finally, the “randomForest” R package was employed to rank the importance of each gene using the random forest classification algorithm.

*PPI network analysis*: We utilized STRING database (https://string‐db.org)^37^ to examine the functional interactions among proteins targeted by the top DEGs identified between the high and low M1 macrophage groups. Cytoscape was applied to develop a PPI network diagram. GENEMANIA (http://genemania.org/search/), a versatile and user‐friendly web interface, was employed to generate hypotheses regarding gene function with high accuracy of prediction, facilitating the analysis of gene lists and the prioritization of genes for subsequent functional assays.^38^ We employed GENEMANIA to construct a gene–gene interaction network, assessing the connections between the top DEGs and their closest counterparts, along with an exploration of their functions.

### 5.3 Single‐cell RNA sequencing

*Collection and preparation of samples*: Fresh breast tissues from two TNBC patients were collected within 30 min of surgery and preserved in sCelLiveTM Tissue Preservation Solution (Singleron Bio Com). The tissues were then washed three times with Hanks Balanced Salt Solution and cut into small pieces measuring 1–2 mm. Subsequently, the tissue fragments were dissociated and digested using 2 mL of GEXSCOPETM dissociation solution (Singleron) at 37°C for 15 min with shaking. After passing the samples through a 40‐μm sterile filter, the filtrate was centrifuged for 5 min at 1000 rpm. Following the disposal of the supernatant, the cells were resuspended in 1 mL of PBS (HyClone) solution. To eliminate any remaining erythrocytes, 2 mL of GEXSCOPETM erythrocyte lysis buffer (Singleron) was added, and the solution was left at 25°C for 10 min. The number of viable cells was determined by microscopic examination following centrifugation at 500 *g* for 5 min. The cells were resuspended in PBS, stained with trypan blue (Sigma), and then centrifuged at 500 g for 5 min for accurate cell counting.

*Sequencing of single‐cell transcriptome libraries*: The scRNA‐seq libraries were generated from the TNBC tissue samples using the GEXSCOPETM Single‐Cell RNA Library Kit (Singleron Biotechnologies) according to the manufacturer's instructions. The libraries were then sequenced using an Illumina Novaseq 6000 platform with 150 bp reads.

*Analysis of raw read data*: Using CeleScope version 1.4.0 (available at https://github.com/singleron‐RD/CeleScope), the raw reads obtained from scRNA‐seq were processed to generate gene expression matrices. Initially, the raw reads were subjected to quality control using Cutadapt v1.17^39^ through the CeleScope pipeline. This step involved trimming poly‐A tail and adapter sequences, effectively removing low‐quality reads. Subsequently, the barcode and unique molecular identifier (UMI) associated with each cell were extracted. In the next stage, the processed reads were aligned to the GRCh38 reference genome (annotated by Ensembl version 92) using STAR v2.6.1a.^40^ The alignment allowed for mapping the reads to their corresponding genomic locations. Gene counts and UMI counts were then obtained using featureCounts version 2.0.1 software,^41^ enabling the quantification of gene expression levels. Finally, expression matrix files were generated, which served as the basis for further analysis in the study.

*Quality control, dimension reduction, and clustering*: To ensure high‐quality data for downstream analysis, we applied several filtering steps. First, we filtered out cells with gene counts below 200, as well as those with gene counts and UMI counts in the top 2%. Additionally, cells with a mitochondrial content exceeding 20% were removed from further analysis. After filtering, a total of 27,351 cells were retained for subsequent analysis, with an average of 2108.319 genes and 8085.971 UMIs per cell. For dimension reduction and clustering, we utilized functions from Seurat v3.1.2.^42^ After normalizing and scaling the gene expressions, we identified the top 2000 variable genes using the FindVariableFeatures function for principal component analysis (PCA). To further separate the cells into distinct clusters, we employed the FindClusters function, which utilized the top 20 principal components. The batch effect between samples was removed using the Harmony algorithm.^43^ To visualize the cells in a two‐dimensional space, we applied the t‐SNE algorithm. This technique allowed for the representation of the high‐dimensional gene expression data in a lower dimensional space, facilitating the visualization of cell clusters.

### 5.4 Developing a model for predicting ICI efficacy using deep learning and multi‐level attention graph neural network

To develop a novel model for predicting ICI efficacy, we employed deep learning algorithms and focused on M1 macrophage and factors associated with the TIME. The IMvigor210 cohort was selected as the training group, while the mUC cohort2, the HNSCC cohort, and SYSMH‐GC cohort served as the validation groups. The MLA‐GNN has shown strong resilience to batch effects by imitating the biological regulatory process and pinpointing biomarkers at the pathway level. This approach significantly improves the precision and effectiveness in prediction tasks involving both transcriptomic and proteomic data.^44^ Through the use of the full‐gradient graph saliency mechanism, the MLA‐GNN has the capacity to reveal clinically meaningful biomarkers at the pathway level, which, although pertinent, often remains undetected by conventional methods.^44^ In this study, the MLA‐GNN was utilized to construct the novel model based on 294 genes^44^ (Table S2). This approach involved identifying the intersection of differential genes at three levels: gene expression, M1 macrophage infiltration, and TIME‐associated factors. The parameter settings for the MLA‐GNN model were detailed in Table S3, and the Python code to construct the novel model using the MLA‐GNN was displayed in Supporting Information. To examine the performance of the model, we used the receiver operating characteristic (ROC) and AUC to estimate its accuracy in predicting ICI efficacy. The ROC curve offers a visual display of the balance between the true positive rate and the false positive rate, allowing for the assessment of the model's predictive capabilities.

### 5.5 Statistical analysis

For categorical variables, comparisons were performed using the chi‐square test or Fisher's exact test, while continuous variables were analyzed using the Mann–Whitney test for binary comparisons, or through one‐way analysis of variance complemented by the Kruskal–Wallis test for multi‐group comparisons. Spearman correlation analysis was used to assess correlation coefficients. OS was estimated with the Kaplan–Meier method and compared using the log‐rank test. The optimal cutoff values for continuous variables in each dataset were determined using the R package “survminer.” A two‐sided *p*‐value < 0.05 was considered statistically significant. Statistical analyses were conducted using R version 4.0.0 (http://www.rproject.org/) and Python 3.10 version software.

## AUTHOR CONTRIBUTIONS

K.Z., H.R.Y., and Y.F.Y. contributed to supervision, conceptualization, and project administration. Y.F.Y., H.Z.C., W.H.O.Y., and J.Z. wrote and revised the manuscript. Y.F.Y., H.Z.C., W.H.O.Y., and J.Z. contributed to data curation. H.H., L.H.M., X.Y.J., T.H.G., Z.H.W., R.C.L., J.Z.H., and H.Q.Y. contributed to data curation and data analysis. All authors have read and approved the final manuscript.

## CONFLICT OF INTEREST STATEMENT

Author Kang Zhang is an Editorial board member of Medcomm. Author Kang Zhang was not involved in the journal's review or decisions related to this manuscript. The other authors declared no conflict of interest.

## ETHICS APPROVAL

The study adhered to the principles outlined in the Declaration of Helsinki and received approval from the ethics committee of Sun Yat‐sen Memorial Hospital, Sun Yat‐sen University (Approval Number: SYSEC‐KY‐KS‐2019‐171‐001).

## Supporting information

Supporting Information

## Data Availability

This study used publicly available data from the IMvigor210 trial, GEO (https://www.ncbi.nlm.nih.gov/geo), and TCGA (https://portal.gdc.cancer.gov/). Data on patients from Sun Yat‐sen Memorial Hospital of Sun Yat‐sen University that were used to support the finding of this study may be released upon application to the corresponding author Kang Zhang and Herui Yao.
