# Supplementary material for: Unraveling the role of M1 macrophage and CXCL9 in predicting immune checkpoint inhibitor efficacy through multicohort analysis and single‐cell RNA sequencing
Source: MedComm (2020). 2024 Mar 1;5(3):e471. doi: 10.1002/mco2.471 (PMC10906808; doi:10.1002/mco2.471)
Supplement: Supplementary file 1 — Supporting Information [file MCO2-5-e471-s001.docx]

**Supplementary Material**

**Unraveling the Role of M1 Macrophage and CXCL9 in Predicting Immune Checkpoint Inhibitor Efficacy through Multicohort Analysis and Single-Cell RNA Sequencing**

**Running title:** **Predicting ICI Efficacy with M1 Macrophage and CXCL9**

Yunfang Yu^1,2#^, Haizhu Chen^2#^, Wenhao Ouyang^2#^, Jin Zeng^3,4#^, Hong Huang^5^, Luhui Mao^2^, Xueyuan Jia^1^, Taihua Guan^4^, Zehua Wang^6^, Ruichong Lin^7^, Zhenjun Huang^2^, Hanqi Yin^8^, Herui Yao^2*^, Kang Zhang^1,4,9*^

**Author Affiliation**

^1^Faculty of Medicine, Macau University of Science and Technology, Taipa, Macao, PR China.

^2^Guangdong Provincial Key Laboratory of Malignant Tumor Epigenetics and Gene Regulation, Department of Medical Oncology, Breast Tumor Centre, Phase I Clinical Trial Centre, Yat-sen Supercomputer Intelligent Medical Joint Research Institute, Sun Yat-sen Memorial Hospital, Sun Yat-sen University, Guangzhou, China.

^3^Faculty of Sustainable Development, Macau University of Science and Technology, Macau, China.

^4^Guangzhou National Laboratory, Guangzhou, China.

^5^School of Medicine, Guilin Medical University, Guilin, China.

^6^Division of Science and Technology, Beijing Normal University-Hong Kong Baptist University United International College, Zhuhai, China.

^7^Faculty of Innovation Engineering, Macau University of Science and Technology, Taipa, Macao, China

^8^South China Institute of Biomedine, Guangzhou, China.

^9^Zhuhai International Eve Center, Zhuhai People'sHospital and the First Affiliated Hospital of Faculty of Medicine, Macau University of Science and Technology and University Hospital, Zhuhai,China.

#Authors contributed equally to this work and considered co-first authors.

*Corresponding authors

Kang Zhang, MD, PhD, Faculty of Medicine, Macau University of Science and Technology, 999078, Taipa, Macao, China. E-mail: kang.zhang@gmail.com;

And

Herui Yao, MD, PhD, Guangdong Provincial Key Laboratory of Malignant Tumor Epigenetics and Gene Regulation, Department of Medical Oncology, Breast Tumor Centre, Phase I Clinical Trial Centre, Sun Yat-sen Memorial Hospital, Sun Yat-sen University, No. 107 Yanjiang West Road, Guangzhou 510120, P. R. China. Email: yaoherui@mail.sysu.edu.cn.

Table of content

[Table S1. Patient characteristics for the included cohorts 3](#_Toc153964225)

[Table S2. The gene panel consisting of 294 genes used for constructing the novel model for predicting clinical outcomes of ICIs. 5](#_Toc153964226)

[Table S3. The training hyper-parameters of MLA-GNN model. 6](#_Toc153964227)

[Figure S1. Inferior overall survival in patients with high M0 and M2 macrophages in the IMvigor210 trial. 7](#_Toc153964228)

[Figure S2. Kaplan-Meier curves illustrating the overall survival based on M1 macrophage infiltration in patients from the TCGA pan-cancer cohort. 8](#_Toc153964229)

[Figure S3 The multivariate analysis for overall survival based on the mUC-cohort2 and the HNSCC-cohort. 9](#_Toc153964230)

[Figure S4. The correlation between M1 macrophage and immune checkpoints in the SYSMH-BC cohort. 10](#_Toc153964231)

[Figure S5 The PPI analysis of the top DEGs between the high and low M1 macrophage groups. 11](#_Toc153964232)

[Figure S6. The correlation between CXCL9 expression and the response to ICI therapy. 12](#_Toc153964233)

[Figure S7. Kaplan-Meier curves illustrating the overall survival based on CXCL9 expression in patients from the TCGA pan-cancer cohort. 13](#_Toc153964234)

[Figure S8. Pathway enrichment analyses based on the DEGs between the CXCL9 high and low expression groups in the IMvigor210 cohort. 14](#_Toc153964235)

[Figure S9. The correlation between CXCL9 expression and the 22 types of immune cells calculated using CIBERSORT analysis in the IMvigor210 cohort. 15](#_Toc153964236)

[Figure S10. The correlation between CXCL9 expression and immune checkpoints. 16](#_Toc153964237)

[Figure S11. Single-cell RNA sequencing of TNBC tissues reveals the top genes expressed in six main cell clusters. TNBC, triple-negative breast cancer. 17](#_Toc153964238)

[Figure S12. The correlation of APOBEC3G expression with M1 macrophage, CXCL9 expression, and immune cells in the IMvigor210 cohort. 18](#_Toc153964239)

[Figure S13. Pathway enrichment analyses based on the DEGs between the APOBEC3G high and low expression groups in the IMvigor210 cohort. 19](#_Toc153964240)

[Figure S14. Comparison of the predictive performance between the new model and TMB or PD-L1. 20](#_Toc153964241)

[Supplementary Methods 21](#_Toc153964242)

# Table S1. Patient characteristics for the included cohorts

| **Cohort** | **Imvigor210 (n=328)** | **GSE176307 (n=88)** | **GSE140901 (n=28)** | **GSE159067 (n=102)** |
| --- | --- | --- | --- | --- |
| **Tumor type** | mUC | mUC | advanced/metastatic HCC | advanced HNSCC |
| **ICI regimen** | atezolizumab | anti-PD-1/PD-L1 therapies | anti-PD-1/PD-L1 therapies | anti-PD-1/PD-L1 therapies |
| **Age (Mean±SD)** | - | 68.74±10.23 | 57.69±11.47 | 62.38±9.19 |
| **Follow up time (Mean±SD) (months)** | 10.25±7.66 | 7.86±6.94 | 74.32±74.33 | 9.60±10.09 |
| **Follow up status, n (%)** |  |  |  |  |
| Alive | 116 (33.3) | 31 (35.2) | 4 (16.7) | 10 (9.8) |
| Dead | 232 (66.7) | 57 (64.8) | 20 (83.3) | 92 (90.2) |
| **Gender, n (%)** |  |  |  |  |
| Male | 272 (78.2) | 55 (62.5) | 22 (91.7) | 83 (81.4) |
| Female | 76 (21.8) | 33 (37.5) | 2 (8.3) | 19 (18.6) |
| **Immunotherapy response, n (%)** |  |  |  |  |
| CR/PR | 68 (19.5) | 16 (18.2) | 6 (25) | 11 (10.8) |
| PD/Stable disease | 230 (66.1) | 72 (81.8) | 18 (75) | 91 (89.2) |
| Unknown | 50 (14.4) | - | - | - |
| **IC level, n (%)** |  |  |  |  |
| IC0 | 97 (27.9) | - | - | - |
| IC1 | 132 (37.9) | - | - | - |
| IC2 | 118 (33.9) | - | - | - |
| Unknown | 1 (0.3) | - | - | - |
| **TC level, n (%)** |  |  |  |  |
| TC0 | 275 (79.0) | - | - | - |
| TC1 | 22 (6.3) | - | - | - |
| TC2 | 50 (14.4) | - | - | - |
| Unknown | 1 (0.3) | - | - | - |
| **T stage, n (%)** |  |  |  |  |
| T1 | - | 5 (5.7) | - | - |
| T2 | - | 59 (67.0) | - | - |
| T3 | - | 18 (20.5) | - | - |
| T4 | - | 4 (4.5) | - | - |
| Unknown | - | 2 (2.3) | - | - |
| **M stage, n (%)** |  |  |  |  |
| M0 | - | 68 (77.2) | - | - |
| M1 | - | 18 (20.5) | - | - |
| Unknown | - | 2 (2.3) | - | - |
| **N stage, n (%)** |  |  |  |  |
| N0 | - | 52 (59.1) | - | - |
| N1 | - | 17 (19.3) | - | - |
| N2 | - | 13 (14.8) | - | - |
| N3 | - | 3 (3.4) | - | - |
| Unknown | - | 3 (3.4) | - | - |

Abbreviation: mUC, metastatic urothelial carcinoma; HCC, hepatocellular carcinoma; HNSCC, head and neck squamous cell carcinomas; ICI, immune checkpoint inhibitor; PD-1, programmed cell death-1; PD-L1, programmed cell death-ligand 1; SD, standard deviation; CR, complete response; PR, partial response; PD, progressive disease; IC, immune cell; TC, tumor cell.

Table S2. The gene panel consisting of 294 genes used for constructing the novel model for predicting clinical outcomes of ICIs.

| **The gene list** | | | | | | | |
| --- | --- | --- | --- | --- | --- | --- | --- |
| ISG15 | FASLG | PARP9 | AIF1 | CCL19 | CD27 | IL21R | HCST |
| TNFRSF9 | RGS1 | PARP15 | HLA-DRA | CCL21 | LAG3 | CORO1A | TYROBP |
| PIK3CD | PTPRC | PARP14 | HLA-DRB1 | SUSD3 | CD4 | ITGAL | MAP4K1 |
| TNFRSF1B | LAX1 | GPR171 | HLA-DQA2 | TRAF1 | C3AR1 | NLRC5 | CD37 |
| PLA2G2D | CR1L | LAMP3 | HLA-DQB2 | IFITM1 | CLEC7A | PSMB10 | IL4I1 |
| C1QA | TRAF3IP3 | RTP4 | HLA-DOB | TRIM22 | KLRK1 | IRF8 | NKG7 |
| C1QC | CMPK2 | JAKMIP1 | TAP2 | OR56B1 | KLRC4 | ITGAE | SIGLEC10 |
| C1QB | RSAD2 | CLNK | PSMB8 | SPI1 | KLRC3 | GP1BA | FPR3 |
| CD52 | SLC8A1 | CD38 | PSMB9 | UBE2L6 | KLRC2 | XAF1 | LILRB4 |
| IFI6 | PLEK | LAP3 | TAP1 | MPEG1 | KLRC1 | ACAP1 | NCR1 |
| LCK | ARHGAP25 | DTHD1 | HLA-DMB | MS4A6A | BIN2 | LGALS9 | USP18 |
| IL12RB2 | VAMP5 | FDCSP | HLA-DMA | MS4A1 | ITGB7 | EVI2B | IGLL5 |
| IFI44L | GNLY | CXCL9 | HLA-DPA1 | CD6 | NCKAP1L | SLFN11 | APOL6 |
| IFI44 | CD8A | CXCL10 | HLA-DPB1 | CD5 | ARHGAP9 | SLFN12L | APOL3 |
| MCOLN2 | ZAP70 | CXCL11 | ETV7 | FERMT3 | IFNG | CCL5 | CSF2RB |
| GBP3 | ARHGAP15 | CXCL13 | FGD2 | CTSW | LYZ | CCL4 | IL2RB |
| GBP1 | CYTIP | HERC6 | THEMIS | PTPRCAP | PLXNC1 | IFI35 | RAC2 |
| GBP2 | IFIH1 | HERC5 | SAMD3 | MYO7A | CMKLR1 | FMNL1 | CYTH4 |
| GBP4 | WIPF1 | TNIP3 | TAGAP | BIRC3 | SELPLG | RGS9 | LGALS2 |
| GBP5 | TTN | DDX60 | AOAH | CARD16 | OAS2 | TMC8 | APOBEC3D |
| GFI1 | CCDC141 | GZMK | MYO1G | CARD17 | OASL | CD7 | APOBEC3G |
| VCAM1 | STAT1 | GZMA | NCF1 | IL10RA | EPSTI1 | SECTM1 | PARVG |
| CD53 | STAT4 | IRF1 | FGL2 | CD3E | LCP1 | CD226 | TYMP |
| PTPN22 | CTLA4 | CD74 | SAMD9L | CD3D | TNFSF13B | SIRPG | KLHDC7B |
| CD2 | ICOS | HAVCR2 | TFEC | CD3G | SLC7A7 | CST7 | SAMSN1 |
| PLEKHO1 | DOCK10 | ITK | GIMAP7 | CRTAM | GZMH | HCK | MX1 |
| CTSS | SP110 | DOCK2 | GIMAP4 | IL2RA | GZMB | SLA2 | UBASH3A |
| FCRL3 | SP140 | LCP2 | GIMAP2 | PRKCQ | GPR65 | SAMHD1 | ITGB2 |
| MNDA | PDCD1 | SERPINB9 | TMEM176B | SFMBT2 | WARS | PPP1R16B |  |
| PYHIN1 | EOMES | BTN3A2 | TLR7 | APBB1IP | RASGRP1 | ZBP1 |  |
| SLAMF8 | CCR8 | BTN2A2 | DOK2 | RASSF4 | B2M | ARRDC5 |  |
| SLAMF6 | CXCR6 | BTN3A1 | ADAMDEC1 | WDFY4 | PSTPIP1 | VAV1 |  |
| SLAMF1 | CCR5 | BTN3A3 | IDO1 | PRF1 | ISG20 | MYO1F |  |
| CD48 | TRAT1 | HLA-A | TOX | SPOCK2 | HAPLN3 | ICAM1 |  |
| SLAMF7 | TIGIT | HLA-E | SLA | IFIT2 | IL32 | RASAL3 |  |
| LY9 | CD80 | HLA-C | CD274 | IFIT3 | NLRC3 | JAK3 |  |
| FCER1G | PLA1A | HLA-B | PDCD1LG2 | IFIT1 | CIITA | IL12RB1 |  |
| CD247 | CD86 | LTB | DDX58 | PIK3AP1 | IGSF6 | IFI30 |  |

# Table S3. The training hyper-parameters of MLA-GNN model.

| **Parameter** | **Value** |
| --- | --- |
| Alpha | 0.2 |
| Batch size | 8 |
| Dropout | 0.2 |
| Final learning rate | 0.1 |
| Gpu numbers | 2 |
| Input dimension | 1 |
| Label dimensions | 2 |
| Lambda_cox | 1 |
| Lambda_nll | 1 |
| Lambda_reg | 0.0003 |
| Lin_input_dim | 720 |
| Learning rate | 0.0005 |
| Linear policy | linear |
| Epochs | 5 |
| Omic dimension | 32 |
| Optimizer type | Adam |
| Patience | 0.0005 |
| Weight_decay | 0.0005 |

Figure S1. Inferior overall survival in patients with high M0 and M2 macrophages in the IMvigor210 trial.


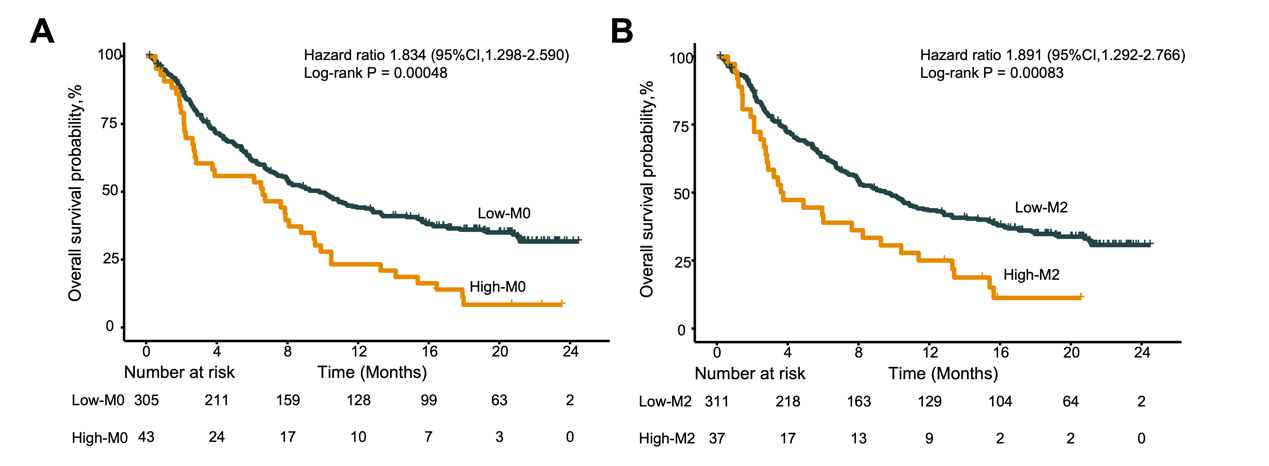


(A) Kaplan-Meier curves of overall survival according to M0 macrophage infiltration; (B) Kaplan-Meier curves of overall survival according to M2 macrophage infiltration.

Figure S2. Kaplan-Meier curves illustrating the overall survival based on M1 macrophage infiltration in patients from the TCGA pan-cancer cohort.

**
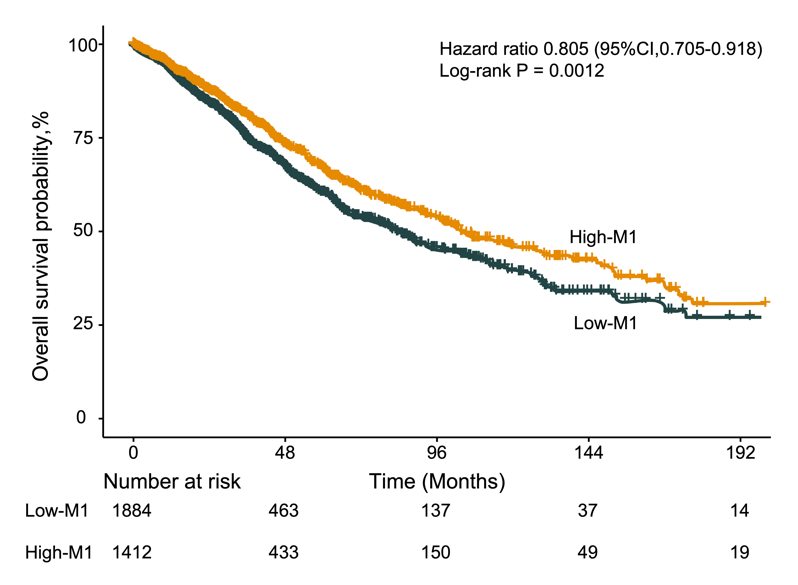
**

Figure S3 The multivariate analysis for overall survival based on the mUC-cohort2 and the HNSCC-cohort.

(A) Forest plot of multivariate analysis for overall survival based on the mUC-cohort; (B) Forest plot of multivariate analysis for overall survival based on the HNSCC-cohort. mUC, metastatic urothelial carcinoma; HNSCC, head and neck squamous cell carcinoma.

Figure S4. The correlation between M1 macrophage and immune checkpoints in the SYSMH-BC cohort.


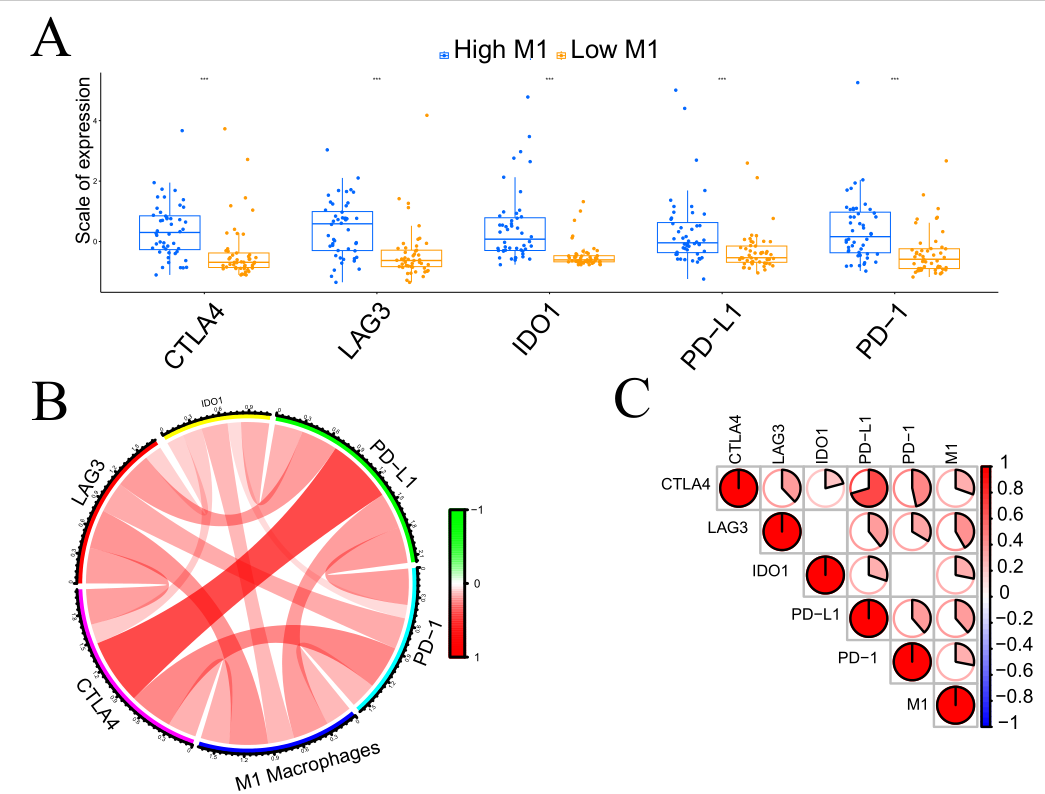


(A) The difference in expression levels of immune checkpoints between the high and low M1 macrophage groups; (B-C) Spearman correlation between M1 macrophage and immune checkpoints. Note: In Figure S3A, ‘***’ represents p-value ≤ 0.001. In Figure S3B, the width and color of the band represents the correlation coefficients. In Figure S3C, the proportion of the pie charts represents the correlation coefficients. BC, breast cancer; CTLA4, cytotoxic T-lymphocyte-associated protein 4; LAG3, lymphocyte-activation gene 3; IDO1, indoleamine 2,3-dioxygenase; PD-L1, programmed cell death-ligand 1; PD-1, anti-programmed cell death-1.

# Figure S5 The PPI analysis of the top DEGs between the high and low M1 macrophage groups.


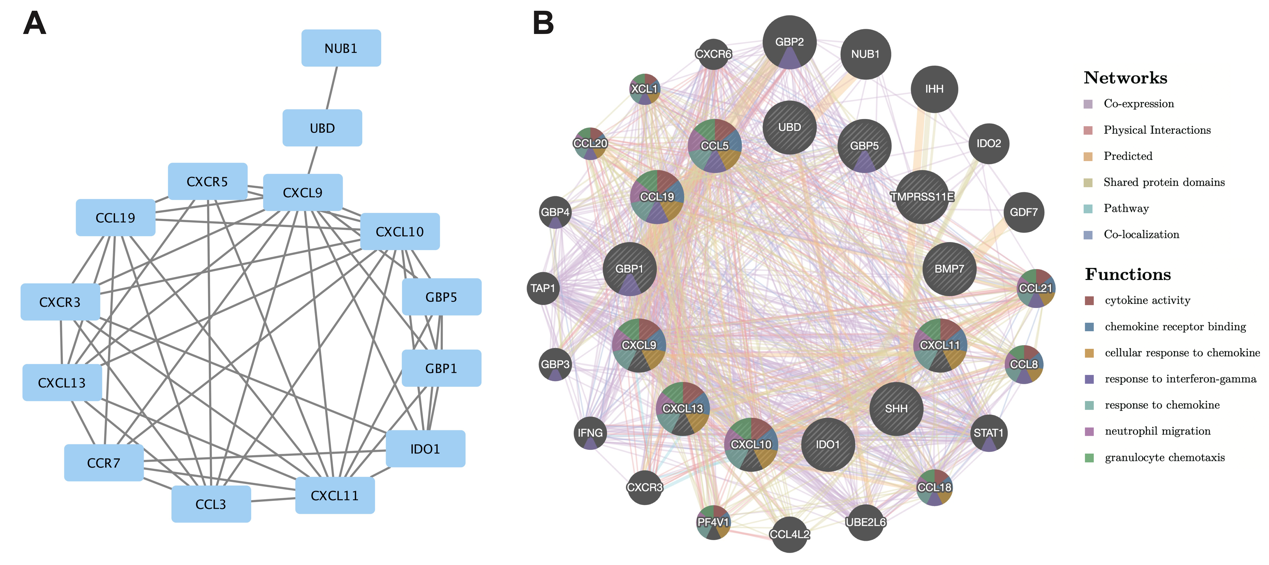


(A) The PPI network diagram of interactions between proteins encoded by the top DEGs between the high and low M1 macrophage groups created by STRING and Cytoscape; (B) The gene-gene interaction network of the top DEGs with the 20 most adjacent genes based on the GeneMANIA database. Each node represents a gene. The color of the linkage of the nodes represents the linkage between the corresponding genes. PPI, Protein-Protein Interaction; DEG, differentially expressed gene.

Figure S6. The correlation between CXCL9 expression and the response to ICI therapy.


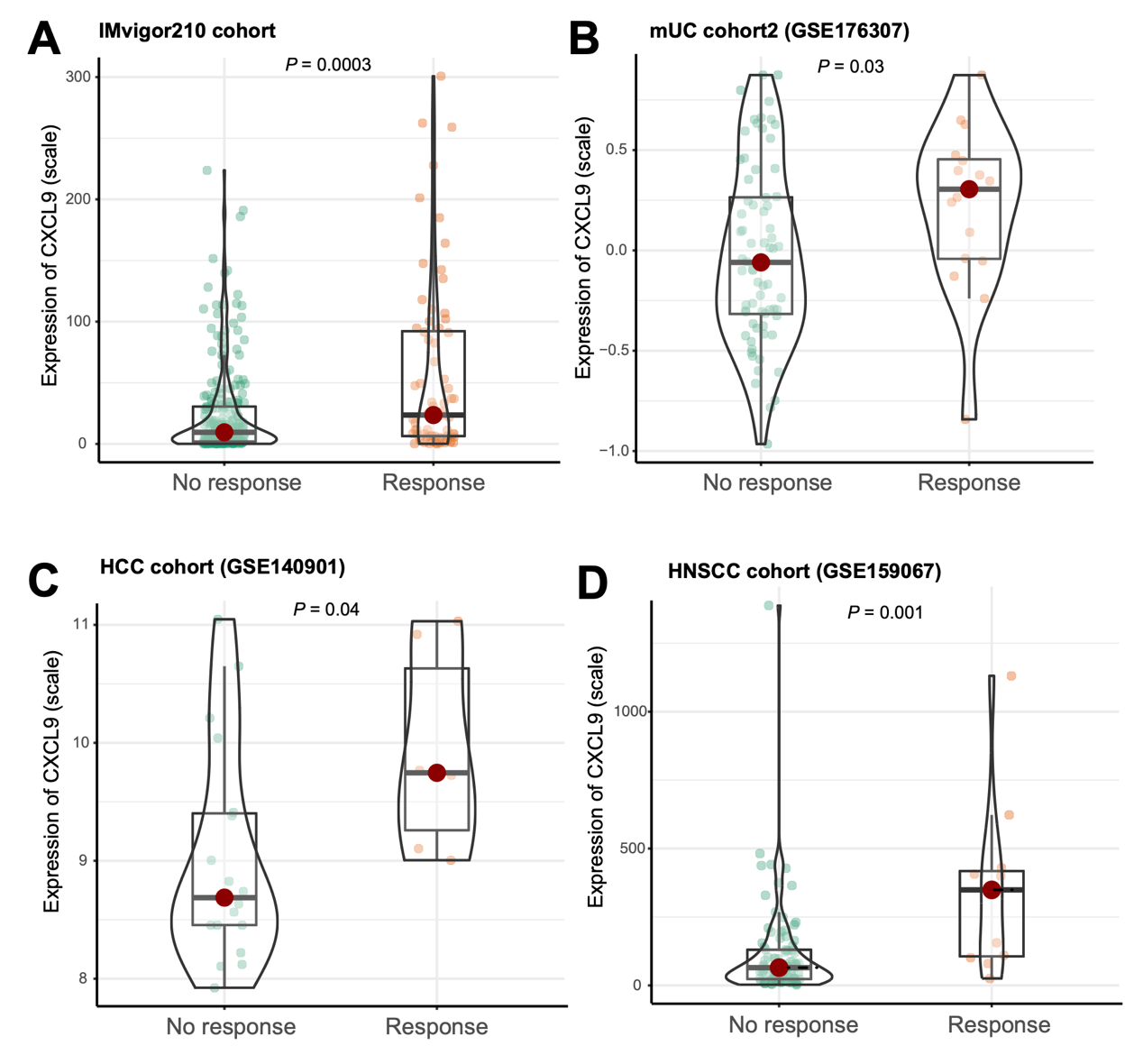


(A-D) The difference in expression levels of CXCL9 between the ICI response group and the ICI non-response group in the IMvigor210 trial (A), mUC cohort2 (B), HCC cohort (C), and HNSCC cohort (D). Note: The significance of the difference was tested by Mann-Whitney test. ICI, immune checkpoint inhibitor; mUC, metastatic urothelial cancer; HCC, hepatocellular carcinoma; HNSCC, head and neck squamous cell carcinoma.

Figure S7. Kaplan-Meier curves illustrating the overall survival based on CXCL9 expression in patients from the TCGA pan-cancer cohort.


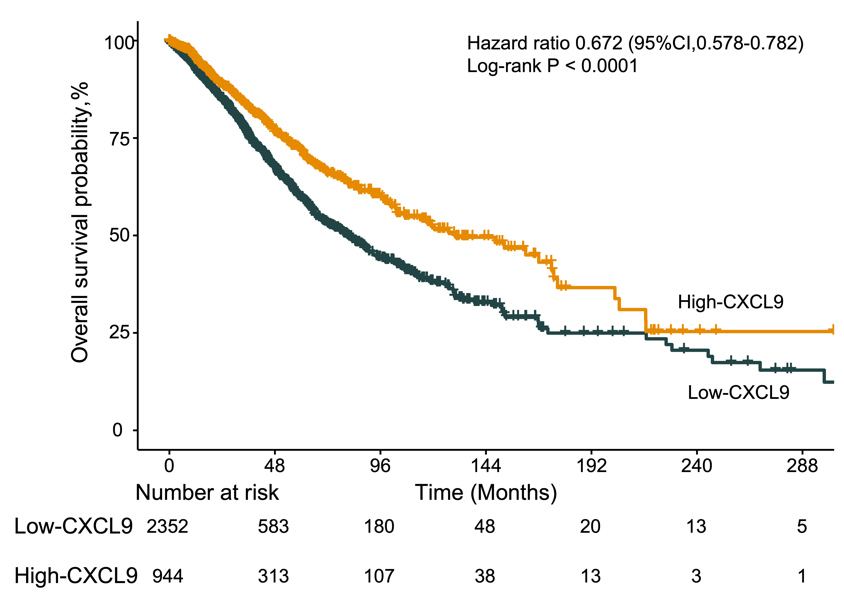


# Figure S8. Pathway enrichment analyses based on the DEGs between the CXCL9 high and low expression groups in the IMvigor210 cohort.

(A) Volcano plot illustrating the DEGs between the high and low expression groups of CXCL9. (B-C) GO (B) and KEGG (C) pathway enrichment analyses conducted on the DEGs. GO, Gene Ontology; KEGG, Kyoto Encyclopedia of Genes and Genomes; DEGs, differentially expressed genes.

Figure S9. The correlation between CXCL9 expression and the 22 types of immune cells calculated using CIBERSORT analysis in the IMvigor210 cohort.


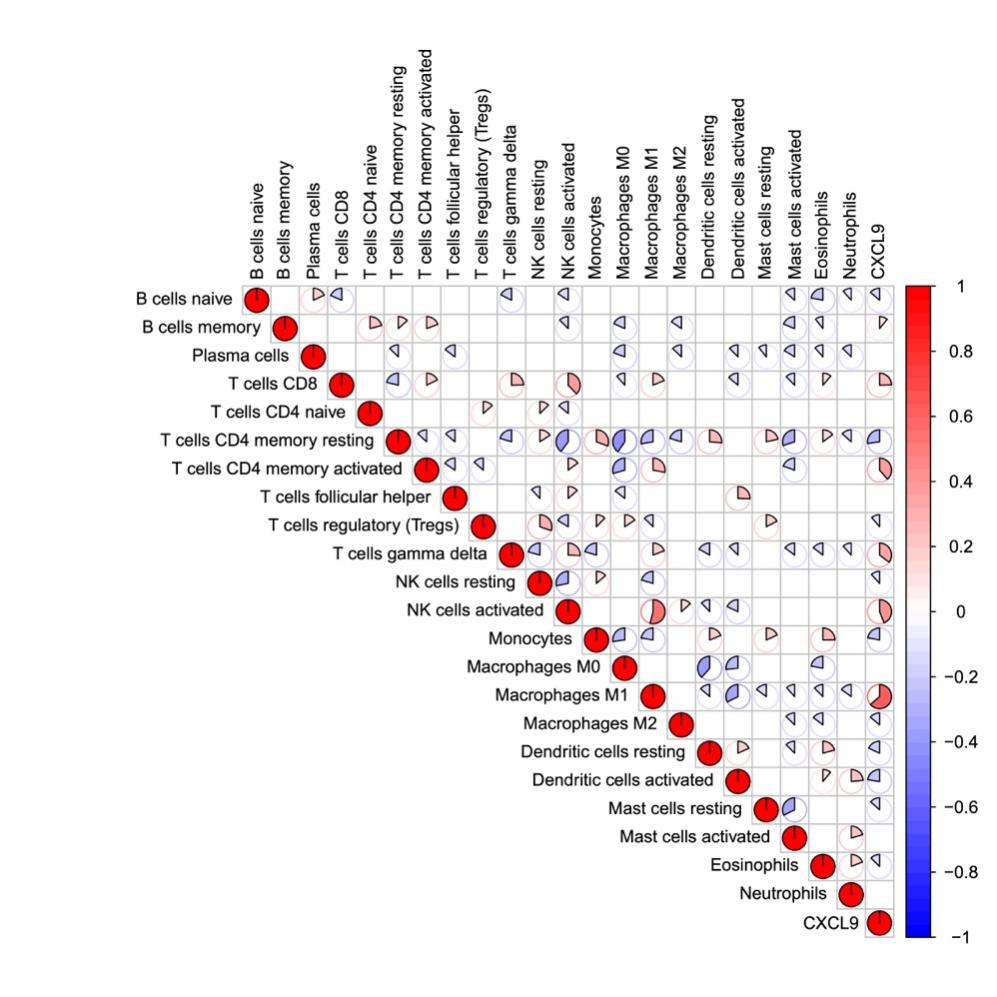


Figure S10. The correlation between CXCL9 expression and immune checkpoints.


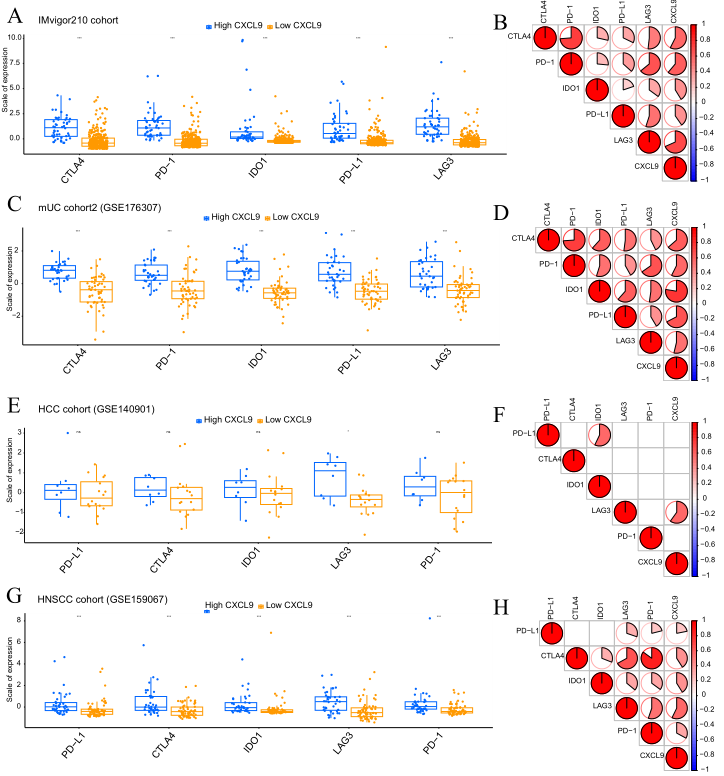


(A, C, E, G) The difference in expression levels of CXCL9 expression between the high and low CXCL9 expression groups in the IMvigor210 (A), mUC cohort2 (C), HCC cohort (E), and HNSCC (G); (B, D, F, H) Spearman correlation between CXCL9 expression and immune checkpoints in the IMvigor210 (B), mUC cohort2 (D), HCC cohort (F), and HNSCC cohort (H). Note: In Figure S7A, 7C,7E, and 7G, ‘*’ represents p-value ≤ 0.05, ‘**’ represents p-value ≤ 0.01, ‘***’ represents p-value ≤ 0.001. In Figure S7B, S7D, S7F, and S7H, the proportion of the pie charts represents the correlation coefficients. PD-1, anti-programmed cell death-1; PD-L1, programmed cell death-ligand 1; CTLA4, cytotoxic T-lymphocyte-associated protein 4; IDO1, indoleamine 2,3-dioxygenase, LAG3, lymphocyte-activation gene 3; mUC, metastatic urothelial cancer; HCC, hepatocellular carcinoma; HNSCC, head and neck squamous cell carcinoma.

Figure S11. Single-cell RNA sequencing of TNBC tissues reveals the top genes expressed in six main cell clusters. TNBC, triple-negative breast cancer.

Figure S12. The correlation of APOBEC3G expression with M1 macrophage, CXCL9 expression, and immune cells in the IMvigor210 cohort.


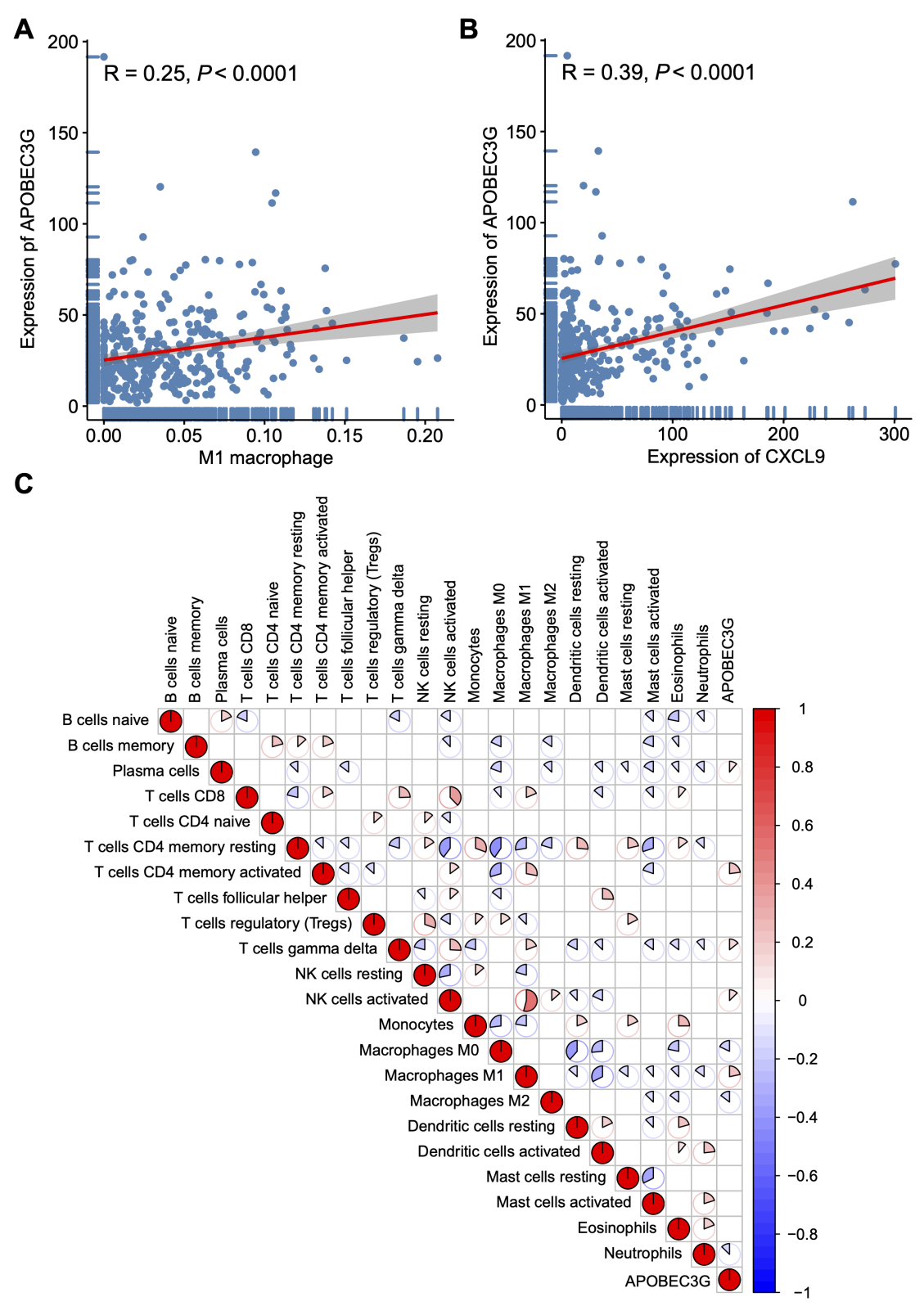


(A) Spearman correlation between APOBEC3G expression and M1 macrophage infiltration; (B) Spearman correlation between APOBEC3G expression and CXCL9 expression; (C) The correlation of APOBEC3G expression with 22 kinds of immune cells calculated by CIBERSORT analysis.

Figure S13. Pathway enrichment analyses based on the DEGs between the APOBEC3G high and low expression groups in the IMvigor210 cohort.

Figure S14. Comparison of the predictive performance between the new model and TMB or PD-L1.

(A) The AUC for ORR prediction of the new model, TMB and PD-L1 in the IMvigor210 cohort; (B) The AUC for ORR prediction of the new model and PD-L1 in the mUC-cohort2. Note: In Figure S14A, the AUC of 0.780 for the novel model was obtained based on patients who had both data on PD-L1 and TMB in the IMvigor210 cohort (n=192). In Figure S14B, the AUC of 0.73 for the novel model was obtained from patients who had data on PD-L1 in the mUC-cohort2 (n=87). Data on TMB was unavailable in the mUC-cohort2, thus the predictive ability of the novel model we constructed was not compared with TMB. TMB, tumor mutational burden; PD-L1, programmed cell death-ligand 1; AUC, area under curve; ORR, objective response rate.

# Supplementary Methods

**Python code to construct the predictive model using the Multi-level Attention Graph Neural Network.**

"""

This is the implementation of Graph Attention Network.

The code is inspired by "https://github.com/Diego999/pyGAT"

"""

import torch

import torch.nn as nn

import torch.nn.functional as F

from torch.optim import Adam

from torch_geometric.nn import global_mean_pool as gap

from torch.nn import init, Parameter

import torch.optim.lr_scheduler as lr_scheduler

from utils import *

class GAT(nn.Module):

def __init__(self, opt, input_dim, omic_dim, label_dim, dropout, alpha):

super(GAT, self).__init__()

self.dropout = dropout

self.act = define_act_layer(act_type=opt.act_type)

self.nhids = [8, 16, 12]

self.nheads = [4, 3, 4]

self.fc_dim = [64, 48, 32]

self.attentions1 = [SpGraphAttentionLayer(

input_dim, self.nhids[0], dropout=dropout, alpha=alpha, concat=True) for _ in range(self.nheads[0])]

for i, attention1 in enumerate(self.attentions1):

self.add_module('attention1_{}'.format(i), attention1)

self.attentions2 = [SpGraphAttentionLayer(

self.nhids[0] * self.nheads[0], self.nhids[1], dropout=dropout, alpha=alpha, concat=True) for _ in

range(self.nheads[1])]

for i, attention2 in enumerate(self.attentions2):

self.add_module('attention2_{}'.format(i), attention2)

self.attentions3 = [SpGraphAttentionLayer(

self.nhids[1] * self.nheads[1], self.nhids[2], dropout=dropout, alpha=alpha, concat=True) for _ in

range(self.nheads[2])]

for i, attention3 in enumerate(self.attentions3):

self.add_module('attention3_{}'.format(i), attention3)

self.dropout_layer = nn.Dropout(p=self.dropout)

# lin_input_dim = self.nhids[0]*self.nheads[0] + self.nhids[1]*self.nheads[1] + self.nhids[2]*self.nheads[2]

lin_input_dim = opt.lin_input_dim

# self.lin1 = torch.nn.Linear(lin_input_dim, lin_dim1)

# self.lin2 = torch.nn.Linear(lin_dim1, label_dim)

self.pool1 = torch.nn.Linear(self.nhids[0] * self.nheads[0], 1)

self.pool2 = torch.nn.Linear(self.nhids[1] * self.nheads[1], 1)

self.pool3 = torch.nn.Linear(self.nhids[2] * self.nheads[2], 1)

fc1 = nn.Sequential(

nn.Linear(lin_input_dim, self.fc_dim[0]),

nn.ELU(),

nn.AlphaDropout(p=self.dropout, inplace=False))

fc2 = nn.Sequential(

nn.Linear(self.fc_dim[0], self.fc_dim[1]),

nn.ELU(),

nn.AlphaDropout(p=self.dropout, inplace=False))

fc3 = nn.Sequential(

nn.Linear(self.fc_dim[1], self.fc_dim[2]),

nn.ELU(),

nn.AlphaDropout(p=self.dropout, inplace=False))

fc4 = nn.Sequential(

nn.Linear(self.fc_dim[2], omic_dim),

nn.ELU(),

nn.AlphaDropout(p=self.dropout, inplace=False))

self.encoder = nn.Sequential(fc1, fc2, fc3, fc4)

self.classifier = nn.Sequential(nn.Linear(omic_dim, label_dim))

self.output_range = Parameter(torch.FloatTensor([6]), requires_grad=False)

self.output_shift = Parameter(torch.FloatTensor([-3]), requires_grad=False)

def forward(self, x, adj, opt):

# print("input shape:", x.shape)

batch = torch.linspace(0, x.size(0) - 1, x.size(0), dtype=torch.long)

batch = batch.unsqueeze(1).repeat(1, x.size(1)).view(-1).cuda()

if opt.cnv_dim == 80:

cnv_feature = torch.mean(x[:, :80, :], dim=-1)

# x = x[:, 80:, :]

x0 = torch.mean(x, dim=-1)

# print("x0:", x0.shape)

x = self.dropout_layer(x)

x = torch.cat([att(x, adj) for att in self.attentions1], dim=-1) # [bs, N, nhid1*nhead1]

x1 = self.pool1(x).squeeze(-1)

# print("x1:", x1.shape)

x = self.dropout_layer(x)

x = torch.cat([att(x, adj) for att in self.attentions2], dim=-1) # [bs, N, nhid2*nhead2]

x2 = self.pool2(x).squeeze(-1)

# print("x2:", x2)

if opt.lin_input_dim == 800 or opt.lin_input_dim == 720:

x = torch.cat([x0, x1, x2], dim=1)

elif opt.lin_input_dim == 320 or opt.lin_input_dim == 240:

if opt.which_layer == 'layer1':

x = x0

elif opt.which_layer == 'layer2':

x = x1

elif opt.which_layer == 'layer3':

x = x2

else:

x = torch.cat([x0, x1, x2], dim=1)

if opt.cnv_dim == 80:

x = torch.cat([cnv_feature, x], dim=1)

GAT_features = x

# print("feature shape:", x.shape)

features = self.encoder(x)

out = self.classifier(features)

fc_features = features

if self.act is not None:

out = self.act(out)

if isinstance(self.act, nn.Sigmoid):

out = out * self.output_range + self.output_shift

return GAT_features, fc_features, out

class GraphAttentionLayer(nn.Module):

def __init__(self, in_features, out_features, dropout, alpha, concat=True):

super(GraphAttentionLayer, self).__init__()

self.dropout = dropout

self.in_features = in_features

self.out_features = out_features

self.alpha = alpha

self.concat = concat

self.W = nn.Parameter(torch.zeros(size=(in_features, out_features)))

nn.init.xavier_uniform_(self.W.data, gain=1.414)

self.a = nn.Parameter(torch.zeros(size=(2 * out_features, 1)))

nn.init.xavier_uniform_(self.a.data, gain=1.414)

self.leakyrelu = nn.LeakyReLU(self.alpha)

self.dropout_layer = nn.Dropout(p=self.dropout)

def forward(self, input, adj):

"""

input: mini-batch input. size: [batch_size, num_nodes, node_feature_dim]

adj: adjacency matrix. size: [num_nodes, num_nodes]. need to be expanded to batch_adj later.

"""

h = torch.matmul(input, self.W) # [bs, N, F]

bs, N, _ = h.size()

a_input = torch.cat([h.repeat(1, 1, N).view(bs, N * N, -1), h.repeat(1, N, 1)], dim=-1).view(bs, N, -1,

2 * self.out_features)

# print("h size:", a_input.shape)

e = self.leakyrelu(torch.matmul(a_input, self.a).squeeze(3))

batch_adj = torch.unsqueeze(adj, 0).repeat(bs, 1, 1)

# print("batch adj size:", batch_adj.shape)

zero_vec = -9e15 * torch.ones_like(e)

attention = torch.where(batch_adj > 0, e, zero_vec)

attention = self.dropout_layer(F.softmax(attention, dim=-1)) # [bs, N, N]

# print("attention shape:", attention.shape)

h_prime = torch.bmm(attention, h) # [bs, N, F]

# print("h_prime:", h_prime.shape)

if self.concat:

return F.elu(h_prime)

else:

return h_prime

def __repr__(self):

return self.__class__.__name__ + ' (' + str(self.in_features) + ' -> ' + str(self.out_features) + ')'

class SpecialSpmmFunction(torch.autograd.Function):

"""Special function for only sparse region backpropataion layer."""

@staticmethod

def forward(ctx, indices, values, shape, b):

assert indices.requires_grad == False

a = torch.sparse_coo_tensor(indices, values, shape)

ctx.save_for_backward(a, b)

ctx.N = shape[0]

return torch.matmul(a, b)

@staticmethod

def backward(ctx, grad_output):

a, b = ctx.saved_tensors

grad_values = grad_b = None

if ctx.needs_input_grad[1]:

grad_a_dense = grad_output.matmul(b.t())

edge_idx = a._indices()[0, :] * ctx.N + a._indices()[1, :]

grad_values = grad_a_dense.view(-1)[edge_idx]

if ctx.needs_input_grad[3]:

grad_b = a.t().matmul(grad_output)

return None, grad_values, None, grad_b

class SpecialSpmm(nn.Module):

def forward(self, indices, values, shape, b):

return SpecialSpmmFunction.apply(indices, values, shape, b)

class SpGraphAttentionLayer(nn.Module):

def __init__(self, in_dim, out_dim, dropout, alpha, concat=True):

super(SpGraphAttentionLayer, self).__init__()

self.alpha = alpha

self.concat = concat

self.W = nn.Parameter(torch.zeros(size=(in_dim, out_dim)), requires_grad=True)

nn.init.xavier_normal_(self.W.data, gain=1.414)

self.a = nn.Parameter(torch.zeros(size=(1, 2 * out_dim)), requires_grad=True)

nn.init.xavier_normal_(self.a.data, gain=1.414)

self.dropout = nn.Dropout(dropout)

self.leakyrelu = nn.LeakyReLU(self.alpha)

self.special_spmm = SpecialSpmm()

def forward(self, input, adj):

dv = 'cuda:0' if input.is_cuda else 'cpu'

b = input.size()[0]

N = input.size()[1]

edge_index = adj._indices()

# First, perform a linear transformation on all features

input = torch.matmul(input, self.W) # [bs, N, F]

# Connect nodes with all neighbors

hidden = torch.cat((input[:, edge_index[0, :], :], input[:, edge_index[1, :], :]), dim=2) # [bs, index, 2*out]

# Obtain scores through vector a

edge_value = torch.exp(-self.leakyrelu(torch.matmul(hidden, self.a.T).squeeze(2))) # 注意力中的分子 [bs, index]

edge_value = self.dropout(edge_value)

for i in range(b):

e_rowsum = self.special_spmm(edge_index, edge_value[i], torch.Size([N, N]), torch.ones(size=(N, 1)).to(dv))

h_prime3 = self.special_spmm(edge_index, edge_value[i], torch.Size([N, N]), input[i])

if i == 0:

h_prime = h_prime3 = h_prime3.div(e_rowsum + torch.Tensor([9e-15]).cuda()).unsqueeze(0)

else:

h_prime3 = h_prime3.div(e_rowsum + torch.Tensor([9e-15]).cuda()).unsqueeze(0)

h_prime = torch.cat((h_prime, h_prime3))

if self.concat:

return F.elu(h_prime)

else:

return h_prime

def define_optimizer(opt, model):

optimizer = None

if opt.optimizer_type == 'adabound':

optimizer = adabound.AdaBound(model.parameters(), lr=opt.lr, final_lr=opt.final_lr)

elif opt.optimizer_type == 'adam':

optimizer = torch.optim.Adam(model.parameters(), lr=opt.lr, betas=(0.9, 0.999), weight_decay=opt.weight_decay)

elif opt.optimizer_type == 'adagrad':

optimizer = torch.optim.Adagrad(model.parameters(), lr=opt.lr, weight_decay=opt.weight_decay,

initial_accumulator_value=0.1)

else:

raise NotImplementedError('initialization method [%s] is not implemented' % opt.optimizer)

return optimizer

def define_reg(model):

for W in model.parameters():

loss_reg = torch.abs(W).sum()

return loss_reg

def define_scheduler(opt, optimizer):

if opt.lr_policy == 'linear':

def lambda_rule(epoch):

lr_l = 1.0 - max(0, epoch + 1) / float(opt.num_epochs + 1)

return lr_l

scheduler = lr_scheduler.LambdaLR(optimizer, lr_lambda=lambda_rule)

elif opt.lr_policy == 'exp':

scheduler = lr_scheduler.ExponentialLR(optimizer, 0.1, last_epoch=-1)

elif opt.lr_policy == 'step':

scheduler = lr_scheduler.StepLR(optimizer, step_size=opt.lr_decay_iters, gamma=0.1)

elif opt.lr_policy == 'plateau':

scheduler = lr_scheduler.ReduceLROnPlateau(optimizer, mode='min', factor=0.2, threshold=0.01, patience=5)

elif opt.lr_policy == 'cosine':

scheduler = lr_scheduler.CosineAnnealingLR(optimizer, T_max=opt.niter, eta_min=0)

else:

return NotImplementedError('learning rate policy [%s] is not implemented', opt.lr_policy)

return scheduler
